# Community Dynamics in Structure and Function of Honey Bee Gut Bacteria in Response to Winter Dietary Shift

**DOI:** 10.1128/mbio.01131-22

**Published:** 2022-08-29

**Authors:** Chenyi Li, Min Tang, Xingan Li, Xin Zhou

**Affiliations:** a College of Food Science and Nutritional Engineering, China Agricultural University, Beijing, People’s Republic of China; b Department of Entomology, College of Plant Protection, China Agricultural University, Beijing, People’s Republic of China; c Key Laboratory for Bee Genetics and Breeding, Jilin Provincial Institute of Apicultural Sciences, Jilin Province, People’s Republic of China; University of California, Irvine

**Keywords:** *Apis mellifera*, overwintering, pollen shortage, *Bartonella*, essential amino acids

## Abstract

Temperate honey bees (Apis mellifera) are challenged by low temperatures and abrupt dietary shifts associated with behavioral changes during winter. Case studies have revealed drastic turnover in the gut microbiota of winter bees, highlighted by the seasonal dominance of a non-core bacterium *Bartonella*. However, neither biological consequence nor underlying mechanism of this microbial turnover is clear. In particular, we ask whether such changes in gut profile are related to winter dietary shift and possibly beneficial to host and associated gut microbiome? Here, we integrated evidences from genomics, metagenomics, and metabolomics in three honey bee subspecies maintained at the same locality of northern China to profile both diversity and functional variations in gut bacteria across seasons. Our results showed that winter dominance of *Bartonella* was shared in all tested honey bee lineages. This seasonal change was likely a consequence of winter dietary shifts characterized by greatly reduced pollen consumption and accumulation of metabolic waste due to restricted excretion. *Bartonella* showed expanded genomic capacity in utilizing more diverse energy substrates, such as converting metabolic wastes lactate and ethanol into pyruvate, an energy source for self-utilization and possibly also for host and other symbionts. Furthermore, *Bartonella* was the only bacterium capable of both producing and secreting tryptophan and phenylalanine, whose metabolic products were detected in bee guts, even though all gut bacteria lacked relevant digestion enzymes. These results thus suggested a possible mechanism where the gut bacteria might benefit the host by supplementing them with essential amino acids lacking in a protein shortage diet.

## INTRODUCTION

The honey bee (Apis mellifera) is an important pollinator that plays a critical ecosystem function in the native range, while also bearing high commercial value in producing bee products (1). As an adaptation to temperate climates, the emergence of long-lived workers (i.e., winter bees) is triggered by pollen resource dwindling (2), and the colony can survive cold winter by forming a thermoregulation cluster (“bee ball”) within the hive, generating heat via intensive vibration of flight muscles (3–6). At the same time, winter bees are confined to the hive without excretion (5), feeding mainly on stored honey (7). In addition to regulating hive temperature, winter bees will also participate in brood rearing in the coming spring (6–8). Hence, the health status of winter bees is vitally important for the whole colony, permitting successful propagation in the year round (9–12).

Honey bees harbor a relatively simple yet crucial gut microbiota, including five core gut bacterial lineages (*Gilliamella*, *Snodgrassella*, *Lactobacillus* Firm 4, *Lactobacillus* Firm 5, and *Bifidobacterium*) (13–15), accounting for >95% of the whole community, and ubiquitous bacteria in low quantity, such as *Frischella*, *Commensalibacter*, and *Bartonella* (15, 16). Increasing evidences have shown diverse beneficial effects of the core gut bacteria on honey bee host, such as immune stimulation (17), pathogenic parasites defense (18–21), detoxification (22), and growth promotion (23, 24). Contrary to extensive studies on core bacteria, the understanding of the impact of non-core bacteria (typically <5% abundance) on honey bees is limited.

The gut bacteria of honey bees are heritable and stable (13, 25), and they are transmitted via social behaviors but are also shaped by diverse factors, such as host genetics (25), antibiotics (26–28), pesticides (29), and food (30–32). In particular, food can drive the differentiation of gut bacterial strains in various animals, from *Drosophila* (33) to humans (34, 35). In bees, pollen diet is critical to the colonization of bee gut bacteria (36), therefore playing a vital role in shaping the gut microbiomes of the honey bees (37) and bumble bees (30). Moreover, the composition and quality of pollen may affect colony health via changing the gut community structure (31).

Given the critical role of honey bee gut bacteria and the impacts of food on both bee health and gut symbionts, an outstanding question remains to be addressed: how do honey bees and gut bacteria cope with the drastic shifts in dietary consumption during winter? During foraging seasons, honey bees consume both pollen and honey as primary food (38). Pollen is rich in nutrients, including ca. 5.9 to 11.5% fat, >20% protein, diverse fatty acids, vitamins, minerals, and antioxidant substances (39, 40), which play vital roles in bee metabolism and hormone regulation (41–43). During winter, without foraging and brood-rearing, bees mainly consume honey (5) but much less pollen (44–46). Honey constitutes primarily sugars, especially glucose and fructose (47, 48), while other nutrients are scarce (48). Therefore, winter bees can be challenged by the shortage of amino acids and lipids. In monophagous and oligophagous insects, such an unbalanced nutrition intake could be complemented by symbionts, a mechanism that effectively increases host fitness and adaptive capacities (49, 50).

Previous studies reported dramatic gut community variations in temperate honey bee colonies over winter, where a non-core bacterium, *Bartonella*, became dominant over core bacteria (36, 51, 52). However, it is not well known whether this microbiota variation is dependent on host lineage or geography, and the underlying cause for the increase of a non-core bacterium was unclear. Nevertheless, given the significantly decreased intake of pollen in temperate honey bees during winter (45), we hypothesize that variations in food structure may be driving the gut microbiome shift.

**FIG 1:**
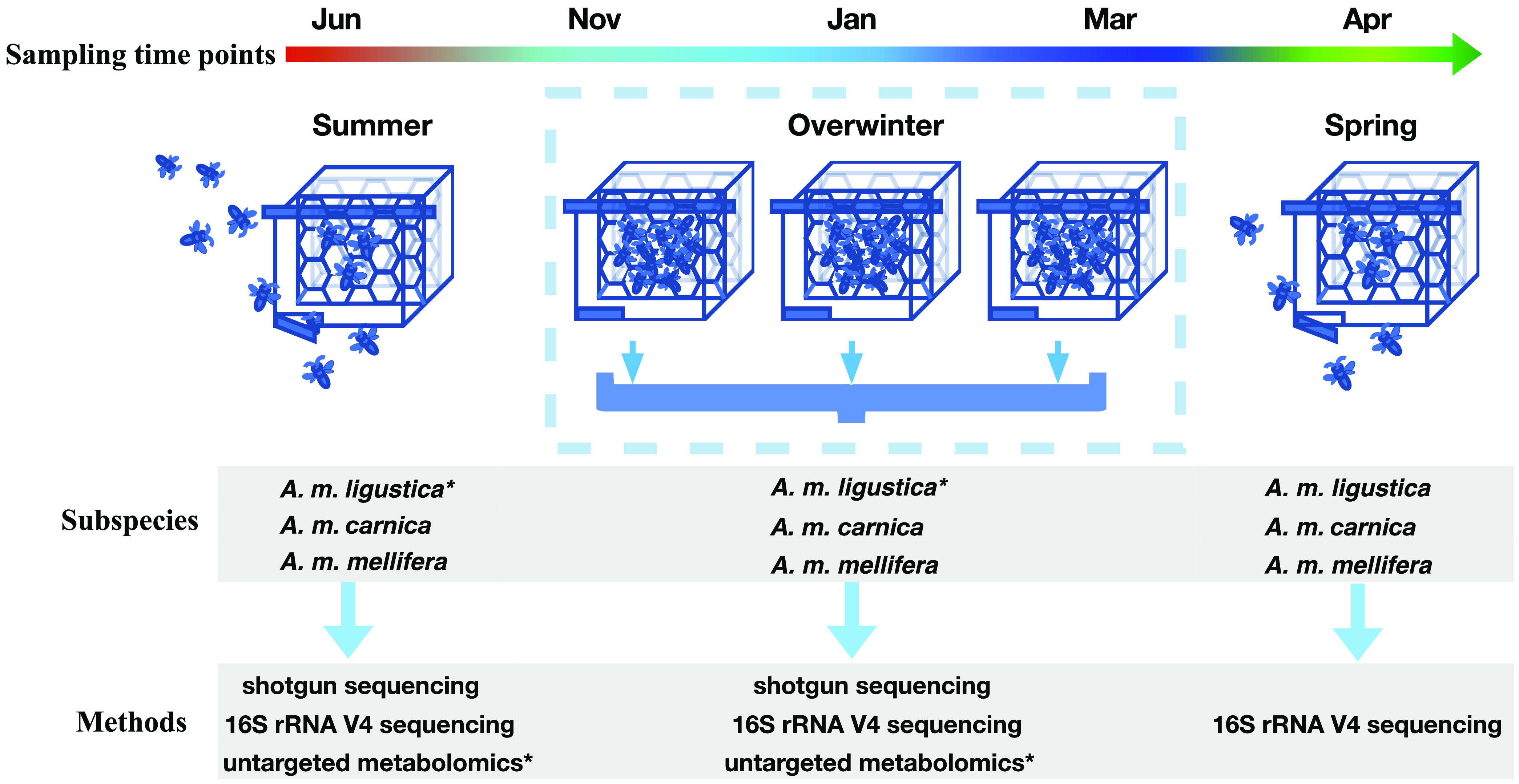
Schematic pipeline for honey bee sampling and analytical methods. Sampling time points: summer (June), early winter (November), midwinter (January), late winter (March), and spring (April). *, Samples analyzed for untargeted metabolomics.

In this study, we sought to understand whether the gut microbiome turnover in winter bees is in concordance with pollen-reduced dietary shift at both community structure and functional levels and whether such variations are common across different honey bee lineages. Furthermore, we examined the possibility whether this seasonal variation might be beneficial to the honey bee host. Using combined evidences from subunit bacterial rRNA (16S) V4 gene fragment sequences, shotgun metagenomics and metabolomics, and multiple *A. mellifera* subspecies reared at the same locality in northeast China (Fig. 1; see also Table S1 in the supplemental material), we showed that seasonal bacterial community change was shared among honey bee lineages, with the non-core bacterium *Bartonella* becoming dominant during winter, while core bacteria remained at decreased abundances. This prominent bacterial turnover was likely due to increased fitness of *Bartonella* under reduced pollen diets because it is capable of utilizing alternative energy substances, e.g., lactate, acetate, and ethanol. Furthermore, comparative genomics revealed that several gut bacteria, especially *Bartonella*, might produce essential amino acids that could have served as a crucial supplement to the honey bee host subject to a protein deficient diet.

10.1128/mbio.01131-22.1 TABLE S1 Honey bees sampling and sequencing. Download Table S1, PDF file, 0.1 MB. Copyright © 2022 Li et al. 2022 Li et al. https://creativecommons.org/licenses/by/4.0/ This content is distributed under the terms of the Creative Commons Attribution 4.0 International license.

## RESULTS

### Significant decline of pollen metabolites in winter bee guts.

**FIG 2:**
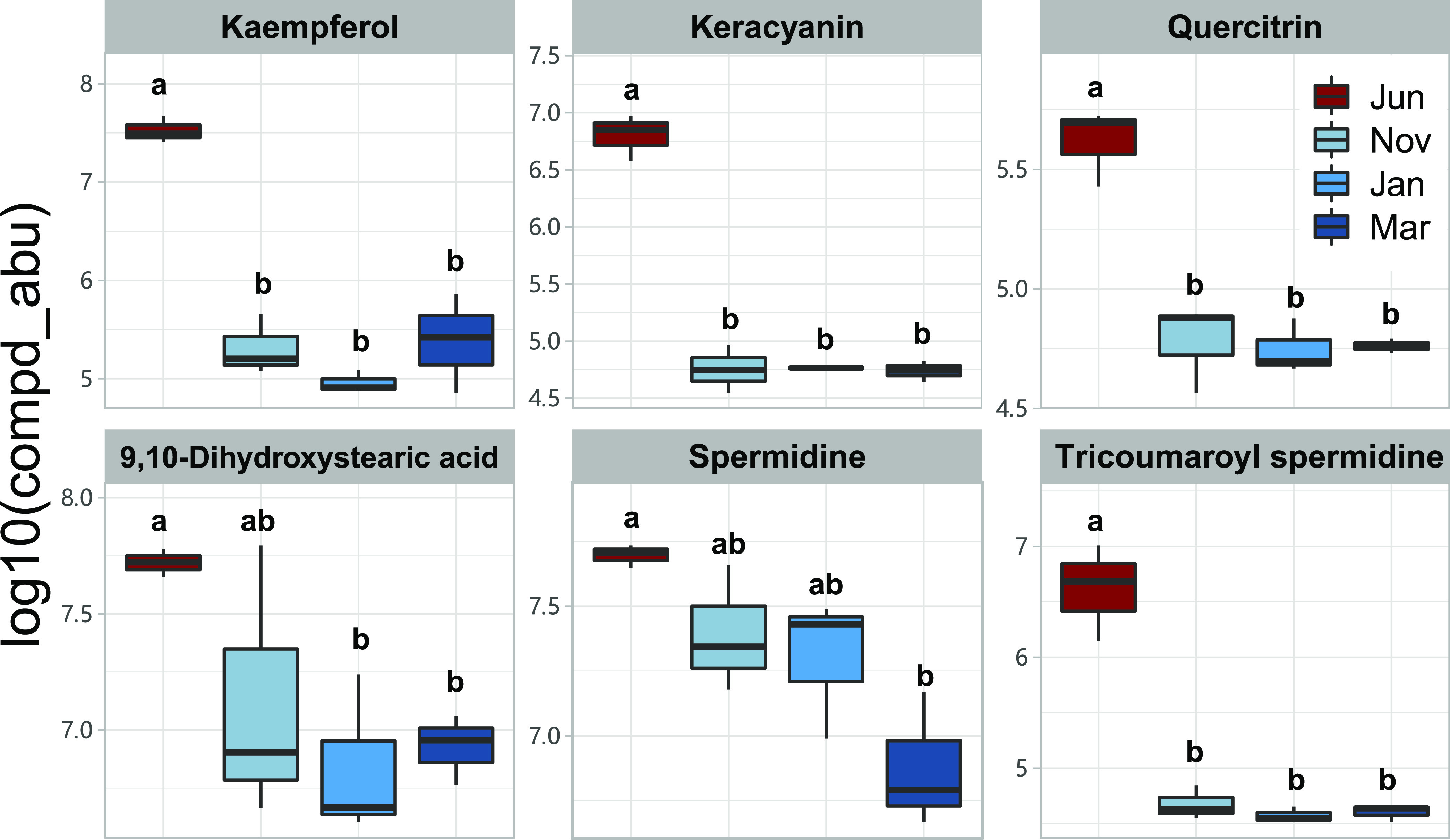
Seasonal variations in pollen-derived substances in honey bee guts. Metabolite variation in honey bee guts in summer (June), early winter (November), midwinter (January), and late winter (March). Letters above each column represent the levels of variation identified in the Wilcoxon test, where different letters indicate significant variations (*P* < 0.05).

Consistent with decreased pollen consumption in winter bees (44, 45, 53), metabolites derived from pollen were significantly reduced in the guts of winter bees. Untargeted metabolomic results for gut samples from four different time points (summer, June; early winter, November; midwinter, January; and late winter, March) revealed that gut metabolites varied significantly throughout the winter phase. In particular, flavonoids (kaempferol, keracyanin, and quercitrin) were all significantly reduced since the beginning of winter (Fig. 2; see also Table S2). The 9,10-dihydroxystearic acid derived from sporopollenin, spermidine, and tricoumaroyl spermidine from exosporium all decreased in the winter bees (Fig. 2; see also Table S2). These results indicated that reduced pollen consumption in the winter bees led to a decrease in relevant nutrients in honey bee guts, which would be expected to influence both the honey bees and their gut microbes.

10.1128/mbio.01131-22.2 TABLE S2 Metabolites involved in metabolism of pollen and amino acids in the honey bee guts. Download Table S2, PDF file, 0.05 MB. Copyright © 2022 Li et al. 2022 Li et al. https://creativecommons.org/licenses/by/4.0/ This content is distributed under the terms of the Creative Commons Attribution 4.0 International license.

### Significant seasonal change of gut microbiota.

**FIG 3:**
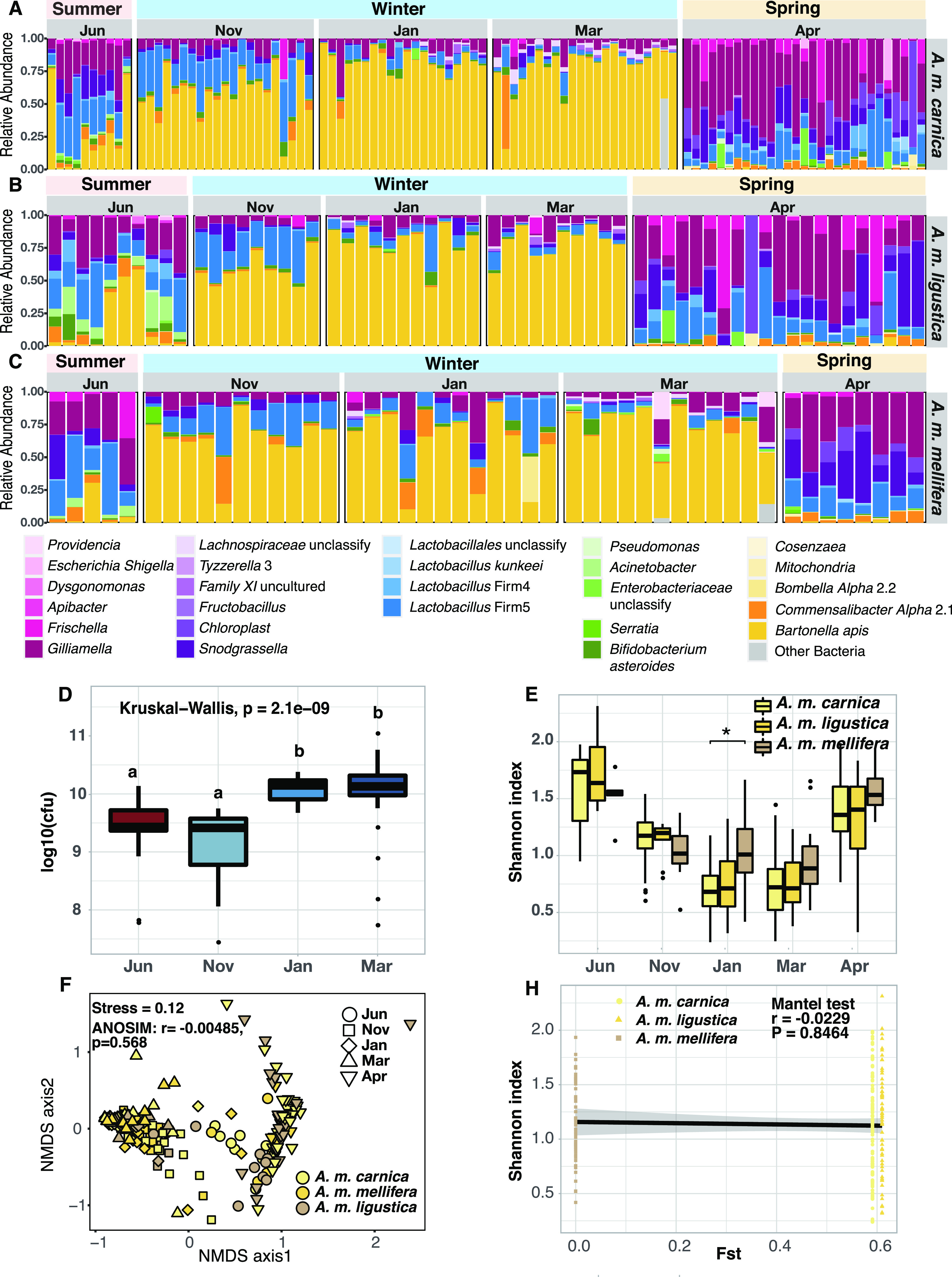
Seasonal variations in gut microbiomes in three honey bee subspecies. (A to C) Relative abundances of gut microbial phylotypes revealed by 16S rRNA V4. (A) *Apis mellifera carnica*; (B) *Apis mellifera ligustica*; (C) *Apis mellifera mellifera*. (D) Variations in live gut bacterial loads in honey bee gut at different times. Gut bacterial load (CFU) counting results for summer (June), early winter (November), midwinter (January), and late winter (March) are shown. (E) Alpha-diversities of gut microbiomes in three honey bee subspecies at different times. (F) NMDS (nonmetric multidimensional scaling) based on the Bray-Curtis dissimilarity determined by 16S rRNA V4. (G) Correlation between the diversity of the gut microbiome and host genetic divergence (*Fst*). *, *P* < 0.05 (Wilcoxon test).

The gut bacterial community of the honey bee displayed seasonal variations, with the most prominent changes condensed in the transition phases of summer-winter and winter-spring (Fig. 3A to C). Throughout winter, the non-core bacterium *Bartonella* became dominant, while the core bacteria decreased conspicuously (Fig. 3A to C; see also Fig. S1A). A significant reduction in alpha diversity in winter bees was supported by both 16S (Kruskal-Wallis, *P* < 0.01) (see Fig. S1B) and shotgun metagenomics (see Fig. S1D). All the samples showed significant temporal clusters in both 16S (ANOSIM, *r* = 0.5597, *P* = 0.001; see Fig. S1C) and shotgun metagenomics results (ANOSIM, *r* = 0.3338, *P* = 0.001; see Fig. S1D). Overall, gut profiles showed clear seasonal turnover characterized by progressive changes.

10.1128/mbio.01131-22.6 FIG S1 Gut community composition and diversity differ between all times. (A) The relative community composition of the gut microbiota was determined by metagenome sequencing. (B and C) Variations in alpha-diversity (B) and NMDS based on Bray-Curtis dissimilarity (C) in microbiota across all times based on 16S rRNA. (D and E) Variations in alpha-diversity (D) and NMDS based on Bray-Curtis dissimilarity in microbiota (E) across all times based on metagenomics. The differences between groups are analyzed by Kruskal-Wallis. Letters above each column represent the levels of variation identified in the Wilcoxon test, where different letters indicate significant variations. Download FIG S1, EPS file, 1.7 MB. Copyright © 2022 Li et al. 2022 Li et al. https://creativecommons.org/licenses/by/4.0/ This content is distributed under the terms of the Creative Commons Attribution 4.0 International license.

In addition to gut bacterial communities, distinguishable differences in the bacterial load in the bee guts were detected. Winter bees do not defecate until spring. Thus, both dead and live bacteria are included in regular metagenomics results. To exclude dead bacteria, we used CFU counts to detect live bacteria. A significant and continuous increase of live bacteria was observed in the bee guts during winter (Kruskal-Wallis, *P* < 0.05) (Fig. 3D). These results demonstrated that both structure and richness of gut microbiota were changed in winter.

### Universal pattern of bacterium shifts in winter bees regardless of host subspecies.

The same microbiota change pattern was observed in all three honey bee subspecies examined in this study: *Apis mellifera carnica*, *Apis mellifera ligustica*, and *Apis mellifera mellifera* (Fig. 3A to C, respectively), which were reared at the same location and were managed following the same protocol. *Bartonella* was dominant in winter, while core bacteria were dominated after winter among all three subspecies. Along the timescale, all subspecies maintained gut microbiota at a significantly reduced alpha-diversity during winter (Fig. 3E). No significant differentiation was detected by Shannon index among hosts, in all but one time point (between *Apis mellifera carnica* and *Apis mellifera ligustica* at midwinter, *P* = 0.006) across the season (Fig. 3E). Samples cannot be differentiated by subspecies (ANOSIM, *r* = –0.00485, *P* = 0.5868; Fig. 3F), and no correlation with honey bee genetics (calculated by *Fst* [54]) was detected (Mantel test, *r* = –0.0229, *P* = 0.8464; Fig. 3G).

### Seasonal function changes of gut microbiota.

**FIG 4:**
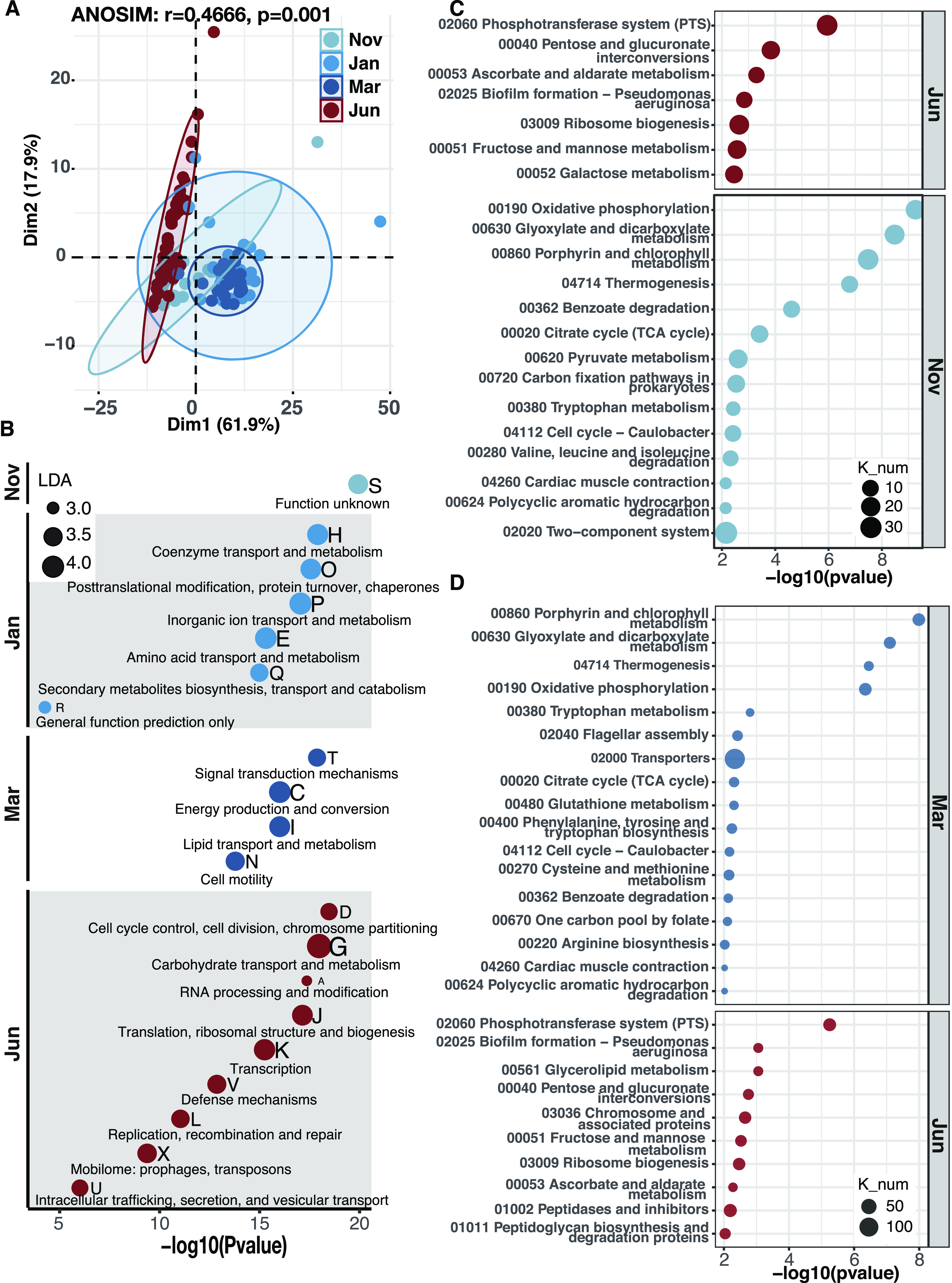
Changes in functional proteins of honey bee gut microbiome over seasons. (A) Principal coordinate analysis plots based on COG categories of different times. (B) LEfSe analysis of functional proteins of honey bee gut microbiota from different times. (C and D) Bubble plots represent enrichments of differential pathways: summer (June) versus early winter (November) (C) and summer (June) versus late winter (March) (D).

Congruent with seasonal changes in bacterial communities, the microbiome function also showed remarkable shifts. The Cluster of Orthologous Groups of protein (COG) profiles were distinguishable and clustered by seasons, within which those sampled from midwinter and late winter (January and March) were prominently distinct from others (Fig. 4A). Although all samples shared the same COG categories, the relative abundance in each COG category was different among seasons. Among the eight metabolism COG categories, the abundance of C, E, H, P, I, and Q categories increased significantly in winter, while that of categories G and F was higher in summer (see Fig. S2). Furthermore, linear discriminant effect size (LEfSe) analyses indicated apparent function transitions between summer and winter bees (Fig. 4B). For example, in midwinter, functional proteins involved in amino-acid transport and metabolism (“E”), coenzyme transport and metabolism (“H”), and inorganic ion transport and metabolism (“P”) were enriched (Fig. 4B). During late winter, the proteins participated in lipid transport and metabolism (“I”) and energy production and conservation (“C”) were featured (Fig. 4B). In contrast, carbohydrate transport and metabolism (“G”) was featured during summer when dietary pollen was available at a regular load.

10.1128/mbio.01131-22.7 FIG S2 Relative abundances of COG annotations related to Metabolism between summer bees and winter bees. Labels: C, energy production and conversion; G, carbohydrate transport and metabolism; E, amino acid transport and metabolism; F, nucleotide transport and metabolism; H, coenzyme transport and metabolism; I, lipid transport and metabolism; P, inorganic ion transport and metabolism; Q, secondary metabolites biosynthesis, transport, and catabolism. ****, *P* < 0.0001 (Wilcoxon test). Download FIG S2, EPS file, 1.5 MB. Copyright © 2022 Li et al. 2022 Li et al. https://creativecommons.org/licenses/by/4.0/ This content is distributed under the terms of the Creative Commons Attribution 4.0 International license.

KEGG annotation results showed that changes in energy metabolism pathways were notable in the early winter bees compared to summer bees, showing enrichments in the oxidative phosphorylation pathway and genes involved in the utilization of pyruvate and dicarboxylic acid (Fig. 4C). The energy metabolism of gut bacteria in summer bees mainly focuses on carbohydrate transport and catabolism, including phosphotransferase system, galactose metabolism, pentose and glucuronate interconversions, and fructose and mannose metabolism (Fig. 4C). In contrast, in winter bees, carbohydrate catabolism declined significantly, while the carboxylic acid catabolism increased.

The microbiome energy functions in late winter phase were characterized by enrichments in the tricarboxylic acid (TCA) cycle pathway and dicarboxylic acid metabolism, while the pyruvate metabolism pathway was not featured (Fig. 4D). Moreover, amino acid metabolism (phenylalanine, tryptophan, and arginine synthesis pathways and cysteine, glutathione, and other metabolic pathways), as well as the metabolism of cofactors and vitamins, was significantly enriched (Fig. 4D). Also, expectedly, functional genes of the phosphotransferase system and carbohydrate metabolism pathways were significantly increased in summer, consistent with the restoration of foraged food.

Among the enrichment pathways in winter (Fig. 4C and D), *Bartonella* was the dominant contributor according to genome comparison among gut bacteria (see Fig. S3B and C). Moreover, the contribution from *Bartonella* continued to increase over winter, from ~70% (early winter; see Fig. S3B) to ~90% (late winter; see Fig. S3C), especially in amino acid metabolism and TCA cycle (see Fig. S3C). In addition, the non-core bacterium *Commensalibacter* was also prevalent in winter bee guts, representing larger contribution than core bacteria (see Fig. S3C). In contrast, the functional profile of summer guts, which had been restored to sugar transport and metabolism, was primarily influenced by core bacterium *Gilliamella*, followed by *Lactobacillus* and *Bifidobacterium* (see Fig. S3A). This pattern suggested that non-core bacteria played an important role in gut microbiome seasonal turnover and that *Bartonella* possessed a competitive advantage over core bacteria in winter bee guts.

10.1128/mbio.01131-22.8 FIG S3 Differential genes of enriched KEGG pathways and proportion of contributions from varied bacterial species. (A) Summer; (B) early winter; (C) late winter. Download FIG S3, TIF file, 1.1 MB. Copyright © 2022 Li et al. 2022 Li et al. https://creativecommons.org/licenses/by/4.0/ This content is distributed under the terms of the Creative Commons Attribution 4.0 International license.

### *Bartonella* was more versatile in energy production.

**FIG 5:**
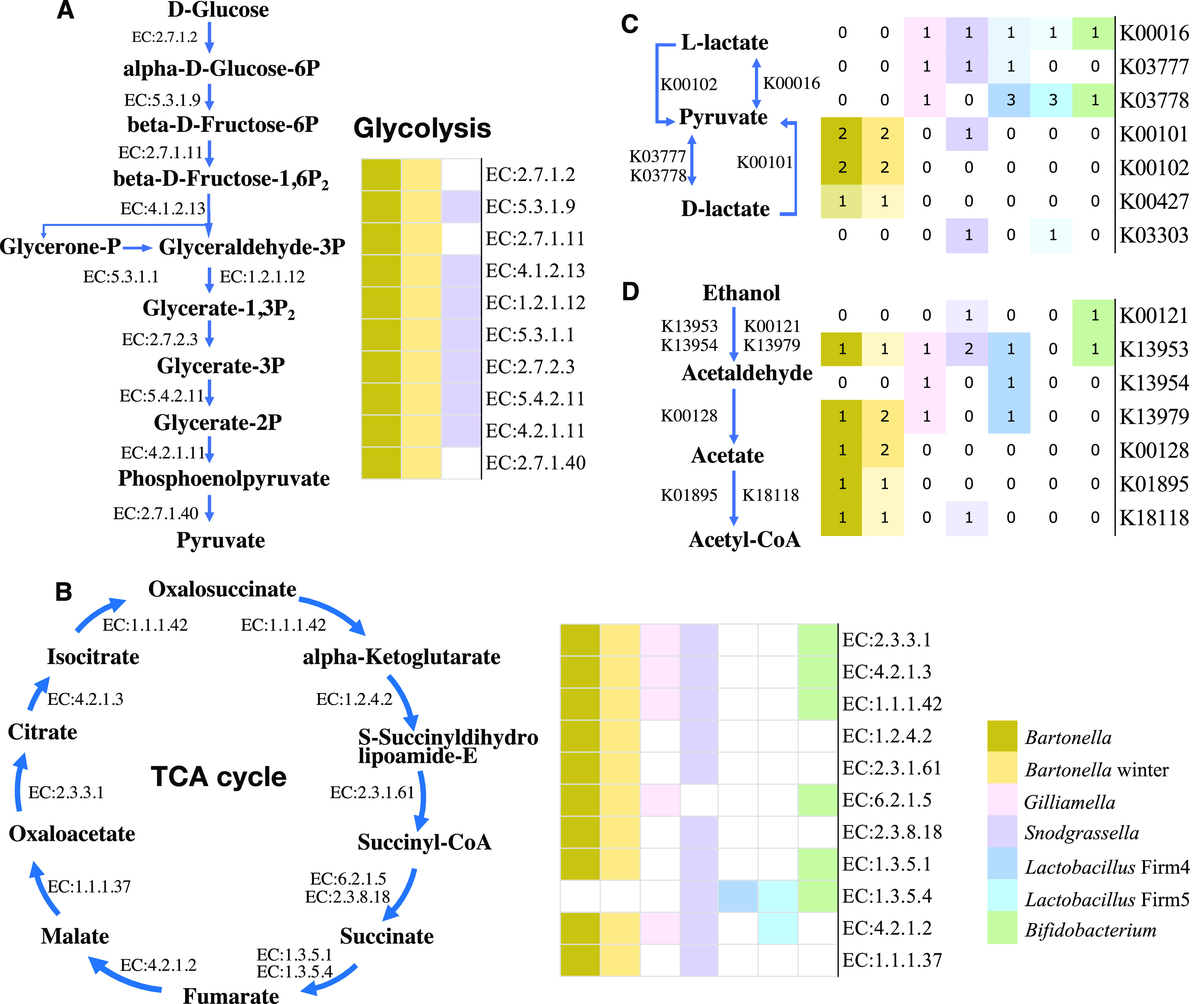
Key genes involved in basic energy pathways (glycolysis and TCA cycle) and waste degradation of *Bartonella* and core bacteria, as suggested by genomes. (A and B) Presence or absence of genes underlying glycolysis and TCA cycle in genomes of *Bartonella* (*n* = 6), a winter *Bartonella* strain (*n* = 1), and core bacteria (*Gilliamella*, *n* = 61; *Snodgrassella*, *n* = 9; *Lactobacillus* Firm 4, *n* = 2; *Lactobacillus* Firm 5, *n* = 13; and *Bifidobacterium*, *n* = 15) from honey bees. (A) Glycolysis; (B) TCA cycle. Colored boxes indicate presence; white boxes indicate absence. (C and D) *Bartonella* and core bacteria varied in copy numbers in genes involved in degradation of fermentation products (lactate, acetate, and ethanol). (C) Lactate; (D) ethanol and acetate. The numbers in the boxes represent gene copy numbers.

Based on enriched functional pathways, TCA cycle pathway was pronounced in late winter (Fig. 4) and *Bartonella* contributed the most (see Fig. S3C). To reveal *Bartonella’s* advantages, comparative analyses of whole genomes were conducted between *Bartonella* and core honey bee gut bacteria (see Table S4). The results showed that *Bartonella* had more diverse pathways in both basic energy production and the utilization of energy sources (Fig. 5A and B). All core bacteria, except *Snodgrassella*, could break down glucose into pyruvate through glycolysis either directly or indirectly. *Snodgrassella*, on the other hand, could conduct the TCA cycle (Fig. 5B), which remedies its deficiency in glycolysis pathway (Fig. 5A). In contrast, only *Bartonella* possessed both pathways (Fig. 5A and B).

10.1128/mbio.01131-22.4 TABLE S4 Genes involved in generating pyruvate and acetyl-CoA of *Bartonella* and core bacteria. Download Table S4, PDF file, 0.1 MB. Copyright © 2022 Li et al. 2022 Li et al. https://creativecommons.org/licenses/by/4.0/ This content is distributed under the terms of the Creative Commons Attribution 4.0 International license.

Congruently, *Bartonella* could utilize more diverse substances for energy production. In addition to glucose, *Bartonella* could also utilize end products of fermentation such as lactate, acetate, and ethanol produced by core bacteria, and independently produce acetyl coenzyme A (acetyl-CoA) (Fig. 5C and D), which can be introduced into TCA cycle or directly used in fatty acid synthesis. For example, *Bartonella* and *Snodgrassella* possessed lactate permease (K00427 or K03303), which could allow extracellular lactate into cells. In addition, *Bartonella* encoded more copies of lactate dehydrogenase (cytochrome) genes than did core bacteria (Fig. 5C), which could catalyze the oxidation of lactate to pyruvate. In addition, only *Bartonella* had the aldehyde dehydrogenase gene (NAD^+^) (K00128), which could convert ethanol into acetate and further into acetyl-CoA (Fig. 5D). Also, *Bartonella* possessed acetyl-CoA synthetase (K01895) for *de novo* synthesizing acetyl-CoA from acetate (Fig. 5D).

### *Bartonella* was capable of supporting host overwintering with nutrients.

Metagenome function profiles showed that amino acid biosynthesis pathways were significantly enhanced in late winter (Fig. 4D), including those of arginine, phenylalanine, tryptophan, cysteine, and methionine, where *Bartonella* had contributed the most (see Fig. S3). Comparative genome analysis revealed that a few essential amino acids, such as phenylalanine and tryptophan, could be synthesized *de novo* by *Bartonella*, *Gilliamella*, and *Snodgrassella* (see Table S3). Interestingly, these bacteria lacked relevant genes to catabolize such amino acids. Compared to *Gilliamella* and *Snodgrassella*, only *Bartonella* encoded the general l-amino acid ABC transporters (*AapP*, *AapQ*, *AapM*, *AapJ*, see Table S3), which were responsible for the extracellular exportation of amino acids.

10.1128/mbio.01131-22.3 TABLE S3 Phenylalanine, tyrosine, and tryptophan biosynthesis and general l-amino acid transport system. Download Table S3, PDF file, 0.1 MB. Copyright © 2022 Li et al. 2022 Li et al. https://creativecommons.org/licenses/by/4.0/ This content is distributed under the terms of the Creative Commons Attribution 4.0 International license.

**FIG 6:**
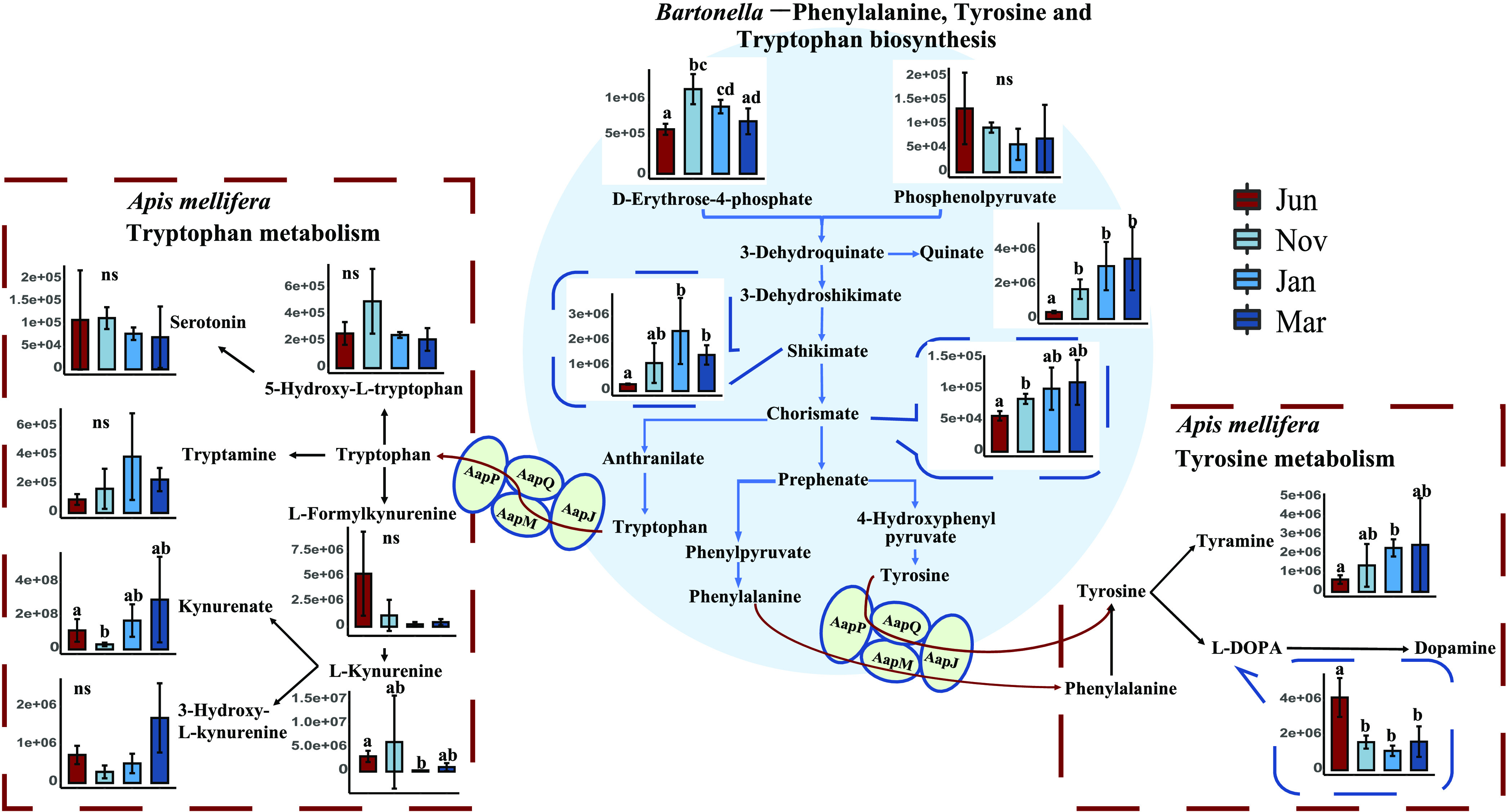
Biosynthesis and degradation of phenylalanine, tryptophan, and tyrosine. The biosynthesis of phenylalanine, tryptophan, and tyrosine by *Bartonella* is shown with a blue background. A simplified process for the host to decompose tryptophan, phenylalanine, and tyrosine is shown in the red box. Plots represent metabolites involved in the metabolism of tryptophan, phenylalanine, and tyrosine. Letters above each column represent the levels of variation identified in a Wilcoxon test, where different letters indicate significant variations. “ns” represents no significance.

The potential bacterial contribution in amino acid synthesis was further supported by our metabolomics results. With a significant reduction in pollen consumption during winter, it was expected that the essential amino acids from pollen would be decreased. However, we detected increased related metabolites in winter bees. The downstream catabolized metabolites of tryptophan and phenylalanine, e.g., tryptamine and tyramine, were significantly elevated. Other catabolites of tryptophan, such as kynurenate and 3-hydroxy-l-kynurenine, were also increased during winter. Similarly, the downstream product of tyrosine (tyramine) derived from the essential amino acid phenylalanine, was increased during winter (Fig. 6; see also Table S2). These products were not likely produced by gut bacteria since bacteria lacked complete gene sets of the catabolic pathways (Fig. 6). Furthermore, as indicated by the pathway enrichment of phenylalanine and tryptophan biosynthesis in gut bacteria, the precursors (e.g., erythrose 4-phosphate) and intermediate substrates of these amino acids (e.g., quinate, shikimate, and chorismate) were significantly increased during winter (Fig. 6; see also Table S2).

## DISCUSSION

For the honey bees, the capability to survive winter is a key adaptive mechanism during its tropic-temperate habitat expansion. However, the behavioral adaptation (forming bee balls and vibrating) and subsequent change in honey bee biology (dietary change and nonexcretion) is a double-edged sword. While it warrants a relative stable colony temperature (6, 55), it also creates a novel challenge to both the host and the gut microbiome. Our study characterized the apparent changes in honey bee gut profile, reflected in both bacterial community structure and function, to understand the underlying mechanism.

### The gut profile variation is in concordance with winter dietary shifts.

It is well known that the majority of pollen-derived nutrition is engaged in honey bee brood-rearing (56) and that the reproduction rhythm of the colony is highly coordinated with the availability of pollen resource (2). For instance, pollen shortage appeared to be a direct food cue (along with environmental changes) that introduced the emergence of winter bees in the fall (2). Although few studies have examined specific changes in colony pollen consumption loads over winter, reports showed that a winter colony might only maintain a pollen hoard equivalent to what was regularly consumed by a summer colony in a single day (45, 46). In the present study, we investigated metabolic variation in winter bee guts. Considering that nutritional variations (e.g., proteins, amino acids, and lipid acids) have been reported between stored and freshly collected pollen (57, 58) and that the winter bees almost exclusively fed on stored pollen, we measured pollen wall components and bioactive constituents (flavonoids) in bee guts, instead of nutrients, as a proxy to quantify pollen consumption change over winter (Fig. 2). Pollen wall components and flavonoids are derived solely from pollen and showed no obvious reduction through storage time (59, 60). Therefore, the reduction in gut flavonoids revealed in our study provided metabolic evidence for pollen consumption decrease in winter bees. On the other hand, the nutrient variations in stored pollen may also have contributed to the reduction of pollen-derived metabolites. Although pollen consumption variation may seem to play a larger role, a systematic quantification of such changes will help to elucidate relative contributions of consumption and nutrient changes to variations in pollen-derived metabolites in winter bee gut. Nevertheless, our study supports that the winter adaptation of the temperate honeybees is associated with dietary tradeoffs between elevated honey and reduced pollen proportions, which in turn triggers a reproduction pause that lasts most of the winter. The shifts in dietary structure show a strong impact on honeybee gut microbiota on both community structure and function profiles (36). As soon as the colony stops foraging, the gut bacterial community switches rapidly to the winter configuration, with declined core bacteria and dominating *Bartonella*, and then returns to the “normal” phase, once food conditions become favorable in the spring. The coordinated reproduction and foraging behaviors of the colony are likely adaptive traits of the host, which is further echoed in the microbiota (Fig. 3A to C). For example, the elevation in lipid transport and metabolism in March may reflect the initiation of the spring brood.

### Winter bacterial turnover is a consistent trait associated with climates but not host lineages.

The characteristic rise of *Bartonella* is notably associated with the temperate winter, during which the experiments were conducted, rather than taxonomic lineage of the honeybees. Previous studies conducted in temperate regions, e.g., Switzerland (36), Canada (61), and Anhui, China (51), showed a similar trend in gut change, which is congruent with findings in the present work. Conversely, seasonal surveys of honey bee gut bacteria on colonies reared in the subtropical regions, such as in Arizona (62) and Colonia, Uruguay (63), revealed much more stabilized gut microbiota throughout the seasons, where *Bartonella* never became dominant. On the other hand, the winter gut profile appeared not to be determined by honeybee lineages. In our study, the three subspecies we examined, reared in sympatry, exhibited a similar trend in gut community variation (Fig. 3). Similarly, multiple hives of local hybrids between *Apis mellifera scutellata*, *Apis mellifera ligustica*, and *Apis mellifera mellifera* shared the subtropical gut profile as described above (63). These results are very different from controlled experiments under laboratory conditions, where host genetics showed a significant indigenous effect on honey bee gut composition (25), suggesting that the seasonal change in food structure has a stronger impact on the gut microbiota in the studied system here.

*Bartonella* is a commensal bacterium in honey bees with high prevalence yet low abundance. However, sporadic elevation of *Bartonella* in summer has also been reported occasionally (52, 64). Possible reasons may include altered diet, temperature change, and behavioral variation of the workers. We expect that systematic tracking of individual biology and behavior may help to elucidate the specific cause of summer abnormality in *Bartonella*. On the other hand, increasing studies have pointed to its correlation to cold conditions. For example, Papp et al. (52) revealed temporal shifts in *Bartonella* with increased abundance were only found at cooler sites. In particular, more studies have shown that *Bartonella* significantly increased during winter (36, 51, 61). Our study suggests, however, that the dominance of *Bartonella* is probably not directly associated with temperature *per se* but rather that the pollen-reduced diet structure caused by seasonal change. Under this special diet structure, the diversified energy pathways may have enabled *Bartonella* to utilize other energy sources, showing superior capability and competitive advantages over core bacteria during winter. Such a diversified capacity may explain the abrupt and consistent increase of *Bartonella* under the temperate winter condition.

### *Bartonella* might be beneficial to host and other gut bacteria.

In addition to the obvious benefit to its own survival, *Bartonella* may be also beneficial to the host and co-occurring gut bacteria. Our genomic inferences indicated that *Bartonella* could generate pyruvate. This result is in congruent with a previous study based on cross-feeding between honey bee gut symbionts, in which the increase of pyruvate was observed in bee guts mono-colonized with *Bartonella* (23). Pyruvate acts as an important energy source, which could be directly utilized by both bacteria and the host (65). In addition, pyruvate can promote the synthesis of trehalose, which is known for its function in improving cold hardiness in insects (66).

Furthermore, our study suggests a new possibility that *Bartonella* might provide essential amino acids to the host. Pollen is the major nutrient food for honey bees, containing a variety of proteins, lipids, carbohydrates, and vitamins (67, 68). The pollen-derived essential amino acids, such as tryptophan and phenylalanine, are precursors of neurotransmitters, which are involved in the regulations of physiological metabolism, nutrient intake, and labor division in honey bees (69). Thus, a shortage of pollen-derived essential amino acids and proteins may have a deleterious impact on bee social behaviors and colony health during winter or even on rear brooding potentials in the spring.

An increasing body of studies have shown that the host could obtain essential amino acids or proteins through symbionts to maintain protein balance (70, 71), such as in termites and brown planthoppers, both of which live on unbalanced diets (72–75). In the present study, our results suggest that the winter diet challenge might be remedied by corresponding changes in gut microbial community and function. Our metagenomics results show that the pathways of amino acid biosynthesis are enriched in late winter, including those of several essential amino acids of honey bees, i.e., phenylalanine, tryptophan, and methionine (Fig. 4), which is primarily contributed by *Bartonella* (see Fig. S3C). Consistently, metabolic intermediates (i.e., quinate, shikimate, and chorismite) produced in bacterial synthesis of phenylalanine and tryptophan have increased during winter, especially in late winter (Fig. 6), which further underlines the role of the gut microbiota as amino acids providers. Interestingly, *Gilliamella*, *Snodgrassella*, and *Bartonella* are all able to independently synthesize tryptophan and phenylalanine and yet are incapable of further decomposition. In principle, these amino acids could be supplied to the host. Congruently, our results reveal elevations in metabolic products derived from degradations of tryptophan and phenylalanine (Fig. 6). These products are most likely generated by the bee host, since only the honey bee possesses the complete pathways. In addition, the genes associated with l-type amino acid transports are only found in the *Bartonella*, allowing them to excrete amino acids out of the cell, while other core bacteria lack these genes. Hence, *Bartonella* may be the major contributor to provide essential amino acids for honey bees during winter, facilitating the winter bees in maintaining health and synthesizing protein for brood.

The interactive responses between host and symbionts under environmental stress have been described in many animal systems, such as in yaks (76, 77), wild mice (78), wild red squirrels (79), ground squirrels (80), stinkbugs (81), and crickets (82). Here, we report that the seasonal dynamics between host food diets and corresponding gut bacterial profiles may reflect coordinated responses of the honey bee and its bacterial symbionts under extreme food and environmental stresses. We showed multiple lines of evidence that are in accordance with the hypothesis that this seasonal variation might involve host-gut bacteria interactions, possibly reflecting adaptation. Admittedly, conclusive evidence is still lacking to provide a definite answer. We expect that further experiments designed specifically to test the impacts of *Bartonella* on the overall fitness of winter bees will help elucidate the evolutionary mechanism underlying the host and its gut symbionts. Finally, in addition to classic evidence where core bacteria can improve the overall honey bee fitness, our study adds yet another insight that honey bees might be able to take advantage of non-core bacteria in extreme cold conditions and are able to restore gut homeostasis when such stresses are removed.

## MATERIALS AND METHODS

### Honey bee sampling.

Honey bee colonies were kept in the apiary of the Jilin Bee Research Institute (located in east longitude 99.58°, northern latitude 25.55°). Sampling was carried out from November 2017 to June 2018. Nurse bees were sampled in June 2018: winter bees were sampled from November 2017 to March 2018; nurse bees transformed from over winter bees (termed “spring bees” here) were sampled in April 2018 (see Table S1). Approximately 40 workers were haphazardly sampled twice a month from each of the 8 hives (2 for *Apis mellifera mellifera*, 4 for *Apis mellifera carnica*, and 2 for *Apis mellifera liguistica*). All honey bee individuals were dissected with sterile forceps to obtain gut tissues, including the mid- and hindguts. Each gut sample was preserved in a 1.5-mL Eppendorf tube, frozen in liquid nitrogen, and later stored at −80°C.

### Untargeted metabolomics.

Guts dissected from winter and summer bees were subject to untargeted metabolomic analyses at Novogen (Beijing, China) (Fig. 1). Each gut (ca. 30 to 70 mg) was individually ground with liquid nitrogen, and the homogenate was resuspended with prechilled 80% methanol and 0.1% formic acid and vortexed. Sample was incubated on ice for 5 min and centrifuged at 15,000 rpm and 4°C for 5 min. The supernatant was diluted to a final concentration of 60% methanol using liquid chromatography-mass spectrometry (LC-MS)-grade water. These samples were subsequently transferred to a new Eppendorf tube with a 0.22 μm filter and then centrifuged at 15,000 rpm and 4°C for 10 min. Finally, the filtrate was used for LC-MS/MS analyses.

The LC-MS/MS analyses were performed using a Vanquish UHPLC system (Thermo Fisher) coupled with an Orbitrap Q Exactive HF-X mass spectrometer (Thermo Fisher). Samples were injected onto a Hyperil Gold column (100 × 2.1 mm, 1.9 μm) using a 16-min linear gradient at a flow rate of 0.2 mL/min. The eluents for the positive polarity mode were eluent A (0.1% formate in water) and eluent B (methanol). The eluents for the negative polarity mode were eluent A (5 mM ammonium acetate [pH 9.0]) and eluent B (methanol). The solvent gradient was set as: 2% B for 1.5 min, an increase to 100% B until 12 min, maintenance for 2 min, and a reduction to 2% B over 0.1 min, followed by holding for 2 min. A Q Exactive HF-X mass spectrometer was operated in the positive/negative polarity mode with a spray voltage of 3.2 kV, a capillary temperature of 320°C, a sheath gas flow rate of 35 arbitrary units, and an auxiliary gas flow rate of 10 arbitrary units. The raw data files generated by UHPLC-MS/MS were processed using Compound Discoverer 3.0 (CD 3.0; Thermo Fisher).

### DNA extraction.

DNA extraction of each batch of samples was conducted within a month after gut dissection. A CTAB (cetyltrimethylammonium bromide)/phenol-based extraction method (24) was used in DNA extraction with minor modification. Briefly, the whole gut was resuspended in a 2-mL tube containing 728 μL of CTAB buffer and 20 μL of 20 mg/mL proteinase K (TransGen Biotech). The mixture was then ground on ice using a TGrinder OST-Y 30, at 8000 rpm for 15 s, and this was repeated three times. Sterile zirconia beads (100 μL [diameter, 0.1 mm]; BioSpec, Bartlesville, OK) and 2 μL of mercaptoethanol were then added to the tubes. Tissues were vortexed using the MOBIO Vortex Genie for 3 min and then lysed by adding 5 μL of RNase A (TransGen Biotech), followed by incubation at 56°C overnight. Lysis was performed using centrifugation at 12,000 × *g* for 5 min, followed by transfer to a new 1.5-mL EP tube after the supernatant was removed. The pellet was mixed with 400 μL of phenol-chloroform-isoamyl alcohol (25:24:1), and the mixture was centrifuged at 12,000 × *g* for 15 min. The supernatant was transferred into a new 1.5-mL tube, and 50 μL of 3 M sodium acetate and 500 μL of isopropanol were added, followed by incubation at −20°C. After centrifugation at 17,000 × *g* for 30 min, the pellets were washed twice with 70% ethanol. Finally, DNA pellets were dissolved in 50 μL of Tris-EDTA buffer (pH 8.0).

### High-throughput sequencing of the 16S rRNA gene fragments.

Amplification of the V4 region of the small subunit (16S) rRNA gene was performed for winter bees (November, January, and March), spring bees (April), and summer bees (June) (Fig. 1). Primers 515F and 806R and a general PCR program were used for 16S V4 amplification. The PCR master mix without DNA template was used as a negative control. Amplicons were sequenced using an Illumina Nova6000 platform with 250-bp paired-end (PE 250) reads, where >30,000 sequences were obtained for each sample. Fastp was used to control the quality of the raw data, and the default parameters were applied to remove reads with a quality value of <20 (83). The program FLASH was used for splicing (-m 15 -x 0.1) (84), and then the merged contigs were imported into QIIME 2 (v2018.8.0) (85). Quality control, denoising, and chimera elimination were performed using DADA2 (86) in QIIME 2 (v2018.8.0). Finally, the representative sequences were classified by a curated SILVA database (https://doi.org/10.5281/zenodo.6772394) for bee gut microbiota (87).

### Shotgun sequencing and *de novo* assembly.

A total of 121 honey bee guts sampled from four time points (November, January, March, and June) were shotgun sequenced (Fig. 1) using a BGI-500 platform with 100 bp paired-end or an Illumina HiSeq X-10 platform with 150 bp paired-end. Raw Illumina reads were filtered using Fastp (v0.13.1 -q 20 -u 10 -w 16) (83), and high-quality reads were subsequently mapped onto the *A. mellifera* genome (GCF_000002195.4) using BWA aln (v0.7.16) (88) to filter out honey bee reads. The remaining reads were *de novo* assembled using Megahit (v1.1.2, k-list 51,61,71,81,91,101,111) (89) for each metagenome, where contigs longer than 500 bp were kept and blast analyzed against the NCBI nr database using DIAMOND (v0.9.22.123, blastx -f 102 -k 1 -e 1e-3) (90) for taxonomic assignment. A customized bacterial database was combined with the NCBI bacterial genomes (37). Assemblies assigned to bacteria were then blast analyzed against the customized bacterial database (blastn -outfmt 6 -e 1e-5 -max_target_seqs 5). Sequences longer than 100 bp were assigned to general bacteria or specific species, when they had a similarity of >30% or >90% to the reference, respectively. For each metagenome, clean reads were mapped onto the assemblies using SOAPaligner (v2.21, -M 4 -l 30 -r 1 -v 5 -m 200) and summarized using the *SOAP.coverage* script (91). Only assemblies with a sequence coverage of >90% were kept for subsequent analyses. The relative abundance of a bacterial species was defined as the number of all bases assigned to the focal species divided by the total number of bases belonging to bacteria in each sample.

### Gene prediction and annotation.

Genes were predicted for bacterial assemblies using MetaGeneMark_linux_64 (92). All predicted genes were clustered using CD-HIT (v4.6.7, -c 0.95 -r 1 -G 1 -g 1 -aS 0.9 -T 24 -M 0) (93, 94) to get a nonredundant gene catalog. Finally, all amino acid sequences of clustered representative genes were annotated using COG category annotation (95) and online KEGG annotation (96). Clean reads were mapped to the nonredundant gene catalog using SOAPaligner, and the gene abundance was summarized using SOAP.coverage (91). The sequence depth of a particular gene was calculated as the total bases mapped onto the focal gene divided by the total bases mapped to any genes in each sample. The differential COG proteins and genes were using LEfSe (97). The pathway enrichment was conducted using a one-sided Fisher exact test in the R package.

### Comparison of bacterial genome.

Core bacterium (100 strains) and *Bartonella* (6 strains) genomes (see Table S5) were downloaded from the NCBI (ftp://ftp.ncbi.nlm.nih.gov/genomes/genbank/bacteria/). *Bartonella* W7133 strain was isolated from winter bee as previously described (98). All the strains were annotated by online KEGG BlastKOALA (96).

10.1128/mbio.01131-22.5 TABLE S5 Bacterial genomes of *Bartonella* and honey bee core bacteria list. Download Table S5, PDF file, 0.1 MB. Copyright © 2022 Li et al. 2022 Li et al. https://creativecommons.org/licenses/by/4.0/ This content is distributed under the terms of the Creative Commons Attribution 4.0 International license.

### Bacterial quantification of gut microbes.

All gut glycerol homogenates were stored in a −80°C freezer for ca. 2 years before plate counting. They were then diluted with phosphate-buffered saline (pH 7.2) and plated on heart infusion agar with 5% sheep blood. Bacterial colonies were subsequently counted after incubation at 35°C and 5% CO_2_ for 48 h.

### Statistical analyses.

All statistical analyses were performed in R version 3.6.0 and visualized using ggplot2 in R. Alpha- and beta-diversities were calculated using the vegan package (99). The Kruskal-Wallis test and Wilcoxon test were used for multigroups, and a *P* value of <0.05 indicated statistical significance.

### Data availability.

Raw data for 16S rRNA and shotgun sequences of winter bees have been deposited under BioProject PRJNA797557, and raw data for 16S rRNA and shotgun sequences of summer bees have been deposited under BioProject numbers PRJNA645267 and PRJNA645015, respectively, in the NCBI database. In-house scripts are available from the corresponding authors upon request.
