# Supplementary material for: Community Dynamics in Structure and Function of Honey Bee Gut Bacteria in Response to Winter Dietary Shift
Source: mBio. 2022 Aug 29;13(5):e01131-22. doi: 10.1128/mbio.01131-22 (PMC9600256; doi:10.1128/mbio.01131-22)
Supplement: TABLE S1 [file mbio.01131-22-s0001.pdf]

Sample	Species	Colony	Sample_time	Sapmle_Location	Latitude_longitude	Sequencing		
B11524	*A. m. carnica*	jlsk3121	Jun	China: Jilin	25.55 N 99.58 E	—	—	untargeted metabolitics
B11525	*A. m. carnica*	jlsk3121	Jun	China: Jilin	25.55 N 99.58 E	—	—	untargeted metabolitics
B11526	*A. m. carnica*	jlsk3121	Jun	China: Jilin	25.55 N 99.58 E	—	—	untargeted metabolitics
W2042	*A. m. carnica*	jlsk3121	Jan	China: Jilin	25.55 N 99.58 E	—	—	untargeted metabolitics
W2044	*A. m. carnica*	jlsk3121	Jan	China: Jilin	25.55 N 99.58 E	—	—	untargeted metabolitics
W2046	*A. m. carnica*	jlsk3121	Jan	China: Jilin	25.55 N 99.58 E	—	—	untargeted metabolitics
W3394	*A. m. carnica*	jlsk3121	Mar	China: Jilin	25.55 N 99.58 E	—	—	untargeted metabolitics
W3396	*A. m. carnica*	jlsk3121	Mar	China: Jilin	25.55 N 99.58 E	—	—	untargeted metabolitics
W3398	*A. m. carnica*	jlsk3121	Mar	China: Jilin	25.55 N 99.58 E	—	—	untargeted metabolitics
W6950	*A. m. carnica*	jlsk3121	Nov	China: Jilin	25.55 N 99.58 E	—	—	untargeted metabolitics
W6952	*A. m. carnica*	jlsk3121	Nov	China: Jilin	25.55 N 99.58 E	—	—	untargeted metabolitics
W6954	*A. m. carnica*	jlsk3121	Nov	China: Jilin	25.55 N 99.58 E	—	—	untargeted metabolitics
B4043	*A. m. carnica*	jlsk3121	November	China: Jilin	25.55 N 99.58 E	metagenome	—	
B4044	*A. m. carnica*	jlsk3121	November	China: Jilin	25.55 N 99.58 E	metagenome	16S rRNA	
B4045	*A. m. carnica*	jlsk3121	November	China: Jilin	25.55 N 99.58 E	metagenome	16S rRNA	
B4046	*A. m. ligustica*	jlyy2060	November	China: Jilin	25.55 N 99.58 E	metagenome	16S rRNA	
B4047	*A. m. ligustica*	jlyy2060	November	China: Jilin	25.55 N 99.58 E	metagenome	16S rRNA	

B4048	*A. m. ligustica*	jlyy2060	November	China: Jilin	25.55 N 99.58 E	metagenome	16S rRNA
B4049	*A. m. mellifera*	jloh5140	November	China: Jilin	25.55 N 99.58 E	metagenome	16S rRNA
B4050	*A. m. mellifera*	jloh5140	November	China: Jilin	25.55 N 99.58 E	metagenome	16S rRNA
B4051	*A. m. mellifera*	jloh5140	November	China: Jilin	25.55 N 99.58 E	metagenome	16S rRNA
B4052	*A. m. carnica*	sk12	November	China: Jilin	25.55 N 99.58 E	metagenome	16S rRNA
B4053	*A. m. carnica*	sk12	November	China: Jilin	25.55 N 99.58 E	metagenome	16S rRNA
B4054	*A. m. carnica*	sk12	November	China: Jilin	25.55 N 99.58 E	metagenome	16S rRNA
B4055	*A. m. carnica*	sk11	November	China: Jilin	25.55 N 99.58 E	metagenome	16S rRNA
B4056	*A. m. carnica*	sk11	November	China: Jilin	25.55 N 99.58 E	metagenome	16S rRNA
B4057	*A. m. carnica*	sk11	November	China: Jilin	25.55 N 99.58 E	metagenome	16S rRNA
B4058	*A. m. ligustica*	jlyy2061	November	China: Jilin	25.55 N 99.58 E	metagenome	16S rRNA
B4059	*A. m. ligustica*	jlyy2061	November	China: Jilin	25.55 N 99.58 E	metagenome	16S rRNA
B4060	*A. m. ligustica*	jlyy2061	November	China: Jilin	25.55 N 99.58 E	metagenome	16S rRNA
B4062	*A. m. mellifera*	jloh5127	November	China: Jilin	25.55 N 99.58 E	metagenome	16S rRNA
B4061	*A. m. mellifera*	jloh5127	November	China: Jilin	25.55 N 99.58 E	metagenome	16S rRNA
B4063	*A. m. mellifera*	jloh5127	November	China: Jilin	25.55 N 99.58 E	metagenome	16S rRNA
B4064	*A. m. carnica*	jlsk3028	November	China: Jilin	25.55 N 99.58 E	metagenome	16S rRNA
B4065	*A. m. carnica*	jlsk3028	November	China: Jilin	25.55 N 99.58 E	metagenome	16S rRNA
B4066	*A. m. carnica*	jlsk3028	November	China: Jilin	25.55 N 99.58 E	metagenome	16S rRNA

W1324	*A. m. carnica*	sk11	January	China: Jilin	25.55 N 99.58 E	metagenome	16S rRNA
W1326	*A. m. carnica*	sk11	January	China: Jilin	25.55 N 99.58 E	metagenome	16S rRNA
W1328	*A. m. carnica*	sk11	January	China: Jilin	25.55 N 99.58 E	metagenome	16S rRNA
W1376	*A. m. carnica*	sk12	January	China: Jilin	25.55 N 99.58 E	metagenome	16S rRNA
W1378	*A. m. carnica*	sk12	January	China: Jilin	25.55 N 99.58 E	metagenome	16S rRNA
W1428	*A. m. mellifera*	jloh5140	January	China: Jilin	25.55 N 99.58 E	metagenome	16S rRNA
W1430	*A. m. mellifera*	jloh5140	January	China: Jilin	25.55 N 99.58 E	metagenome	16S rRNA
W1432	*A. m. mellifera*	jloh5140	January	China: Jilin	25.55 N 99.58 E	metagenome	16S rRNA
W1492	*A. m. mellifera*	jloh5127	January	China: Jilin	25.55 N 99.58 E	metagenome	16S rRNA
W1494	*A. m. mellifera*	jloh5127	January	China: Jilin	25.55 N 99.58 E	metagenome	16S rRNA
W1496	*A. m. mellifera*	jloh5127	January	China: Jilin	25.55 N 99.58 E	metagenome	16S rRNA
W1570	*A. m. carnica*	jlsk3121	January	China: Jilin	25.55 N 99.58 E	metagenome	16S rRNA
W1574	*A. m. carnica*	jlsk3121	January	China: Jilin	25.55 N 99.58 E	metagenome	16S rRNA
W1576	*A. m. carnica*	jlsk3121	January	China: Jilin	25.55 N 99.58 E	metagenome	16S rRNA
W1632	*A. m. carnica*	jlsk3028	January	China: Jilin	25.55 N 99.58 E	metagenome	16S rRNA
W1634	*A. m. carnica*	jlsk3028	January	China: Jilin	25.55 N 99.58 E	metagenome	16S rRNA
W1636	*A. m. carnica*	jlsk3028	January	China: Jilin	25.55 N 99.58 E	metagenome	—
W1698	*A. m. ligustica*	jlyy2060	January	China: Jilin	25.55 N 99.58 E	metagenome	16S rRNA
W1702	*A. m. ligustica*	jlyy2060	January	China: Jilin	25.55 N 99.58 E	metagenome	16S rRNA

W1712	*A. m. ligustica*	jlyy2060	January	China: Jilin	25.55 N 99.58 E	metagenome	16S rRNA
W1786	*A. m. ligustica*	jlyy2061	January	China: Jilin	25.55 N 99.58 E	metagenome	16S rRNA
W1788	*A. m. ligustica*	jlyy2061	January	China: Jilin	25.55 N 99.58 E	metagenome	16S rRNA
W1790	*A. m. ligustica*	jlyy2061	January	China: Jilin	25.55 N 99.58 E	metagenome	16S rRNA
W3858	*A. m. carnica*	sk11	March	China: Jilin	25.55 N 99.58 E	metagenome	16S rRNA
W3862	*A. m. carnica*	sk11	March	China: Jilin	25.55 N 99.58 E	metagenome	16S rRNA
W3864	*A. m. carnica*	sk11	March	China: Jilin	25.55 N 99.58 E	metagenome	16S rRNA
W3942	*A. m. carnica*	sk12	March	China: Jilin	25.55 N 99.58 E	metagenome	16S rRNA
W3944	*A. m. carnica*	sk12	March	China: Jilin	25.55 N 99.58 E	metagenome	16S rRNA
W3946	*A. m. carnica*	sk12	March	China: Jilin	25.55 N 99.58 E	metagenome	16S rRNA
W4194	*A. m. carnica*	jlsk3121	March	China: Jilin	25.55 N 99.58 E	metagenome	16S rRNA
W4196	*A. m. carnica*	jlsk3121	March	China: Jilin	25.55 N 99.58 E	metagenome	16S rRNA
W4198	*A. m. carnica*	jlsk3121	March	China: Jilin	25.55 N 99.58 E	metagenome	16S rRNA
W4268	*A. m. carnica*	jlsk3028	March	China: Jilin	25.55 N 99.58 E	metagenome	16S rRNA
W4272	*A. m. carnica*	jlsk3028	March	China: Jilin	25.55 N 99.58 E	metagenome	16S rRNA
W4274	*A. m. carnica*	jlsk3028	March	China: Jilin	25.55 N 99.58 E	metagenome	16S rRNA
W4352	*A. m. ligustica*	jlyy2060	March	China: Jilin	25.55 N 99.58 E	metagenome	16S rRNA
W4354	*A. m. ligustica*	jlyy2060	March	China: Jilin	25.55 N 99.58 E	metagenome	16S rRNA
W4356	*A. m. ligustica*	jlyy2060	March	China: Jilin	25.55 N 99.58 E	metagenome	16S rRNA

W4402	*A. m. ligustica*	jlyy2061	March	China: Jilin	25.55 N 99.58 E	metagenome	16S rRNA
W4408	*A. m. ligustica*	jlyy2061	March	China: Jilin	25.55 N 99.58 E	metagenome	16S rRNA
W4410	*A. m. ligustica*	jlyy2061	March	China: Jilin	25.55 N 99.58 E	metagenome	16S rRNA
W4474	*A. m. mellifera*	jloh5127	March	China: Jilin	25.55 N 99.58 E	metagenome	16S rRNA
W4478	*A. m. mellifera*	jloh5127	March	China: Jilin	25.55 N 99.58 E	metagenome	16S rRNA
W4480	*A. m. mellifera*	jloh5127	March	China: Jilin	25.55 N 99.58 E	metagenome	16S rRNA
W4526	*A. m. mellifera*	jloh5140	March	China: Jilin	25.55 N 99.58 E	metagenome	16S rRNA
W4528	*A. m. mellifera*	jloh5140	March	China: Jilin	25.55 N 99.58 E	metagenome	16S rRNA
W4530	*A. m. mellifera*	jloh5140	March	China: Jilin	25.55 N 99.58 E	metagenome	16S rRNA
W0024	*A. m. mellifera*	jloh5140	November	China: Jilin	25.55 N 99.58 E	—	16S rRNA
W0026	*A. m. mellifera*	jloh5140	November	China: Jilin	25.55 N 99.58 E	—	16S rRNA
W0030	*A. m. mellifera*	jloh5140	November	China: Jilin	25.55 N 99.58 E	—	16S rRNA
W0080	*A. m. ligustica*	jlyy2061	November	China: Jilin	25.55 N 99.58 E	—	16S rRNA
W0082	*A. m. ligustica*	jlyy2061	November	China: Jilin	25.55 N 99.58 E	—	16S rRNA
W0084	*A. m. ligustica*	jlyy2061	November	China: Jilin	25.55 N 99.58 E	—	16S rRNA
W0147	*A. m. ligustica*	jlyy2060	November	China: Jilin	25.55 N 99.58 E	—	16S rRNA
W0153	*A. m. ligustica*	jlyy2060	November	China: Jilin	25.55 N 99.58 E	—	16S rRNA
W0161	*A. m. ligustica*	jlyy2060	November	China: Jilin	25.55 N 99.58 E	—	16S rRNA
W0243	*A. m. mellifera*	jloh5127	November	China: Jilin	25.55 N 99.58 E	—	16S rRNA

W0245	*A. m. mellifera*	jloh5127	November	China: Jilin	25.55 N 99.58 E	—	16S rRNA
W0251	*A. m. mellifera*	jloh5127	November	China: Jilin	25.55 N 99.58 E	—	16S rRNA
W0290	*A. m. carnica*	jlsk3121	November	China: Jilin	25.55 N 99.58 E	—	16S rRNA
W0292	*A. m. carnica*	jlsk3121	November	China: Jilin	25.55 N 99.58 E	—	16S rRNA
W0294	*A. m. carnica*	jlsk3121	November	China: Jilin	25.55 N 99.58 E	—	16S rRNA
W0353	*A. m. carnica*	sk11	November	China: Jilin	25.55 N 99.58 E	—	16S rRNA
W0355	*A. m. carnica*	sk11	November	China: Jilin	25.55 N 99.58 E	—	16S rRNA
W0357	*A. m. carnica*	sk11	November	China: Jilin	25.55 N 99.58 E	—	16S rRNA
W0401	*A. m. carnica*	jlsk3028	November	China: Jilin	25.55 N 99.58 E	—	16S rRNA
W0403	*A. m. carnica*	jlsk3028	November	China: Jilin	25.55 N 99.58 E	—	16S rRNA
W0419	*A. m. carnica*	jlsk3028	November	China: Jilin	25.55 N 99.58 E	—	16S rRNA
W0466	*A. m. carnica*	sk12	November	China: Jilin	25.55 N 99.58 E	—	16S rRNA
W0468	*A. m. carnica*	sk12	November	China: Jilin	25.55 N 99.58 E	—	16S rRNA
W0490	*A. m. carnica*	sk12	November	China: Jilin	25.55 N 99.58 E	—	16S rRNA
W1330	*A. m. carnica*	sk11	January	China: Jilin	25.55 N 99.58 E	—	16S rRNA
W1332	*A. m. carnica*	sk11	January	China: Jilin	25.55 N 99.58 E	—	16S rRNA
W1334	*A. m. carnica*	sk11	January	China: Jilin	25.55 N 99.58 E	—	16S rRNA
W1380	*A. m. carnica*	sk12	January	China: Jilin	25.55 N 99.58 E	—	16S rRNA
W1382	*A. m. carnica*	sk12	January	China: Jilin	25.55 N 99.58 E	—	16S rRNA

W1384	*A. m. carnica*	sk12	January	China: Jilin	25.55 N 99.58 E	—	16S rRNA
W1434	*A. m. mellifera*	jloh5140	January	China: Jilin	25.55 N 99.58 E	—	16S rRNA
W1436	*A. m. mellifera*	jloh5140	January	China: Jilin	25.55 N 99.58 E	—	16S rRNA
W1438	*A. m. mellifera*	jloh5140	January	China: Jilin	25.55 N 99.58 E	—	16S rRNA
W1498	*A. m. mellifera*	jloh5127	January	China: Jilin	25.55 N 99.58 E	—	16S rRNA
W1500	*A. m. mellifera*	jloh5127	January	China: Jilin	25.55 N 99.58 E	—	16S rRNA
W1502	*A. m. mellifera*	jloh5127	January	China: Jilin	25.55 N 99.58 E	—	16S rRNA
W1578	*A. m. carnica*	jlsk3121	January	China: Jilin	25.55 N 99.58 E	—	16S rRNA
W1580	*A. m. carnica*	jlsk3121	January	China: Jilin	25.55 N 99.58 E	—	16S rRNA
W1582	*A. m. carnica*	jlsk3121	January	China: Jilin	25.55 N 99.58 E	—	16S rRNA
W1638	*A. m. carnica*	jlsk3028	January	China: Jilin	25.55 N 99.58 E	—	16S rRNA
W1640	*A. m. carnica*	jlsk3028	January	China: Jilin	25.55 N 99.58 E	—	16S rRNA
W1642	*A. m. carnica*	jlsk3028	January	China: Jilin	25.55 N 99.58 E	—	16S rRNA
W1694	*A. m. ligustica*	jlyy2060	January	China: Jilin	25.55 N 99.58 E	—	16S rRNA
W1696	*A. m. ligustica*	jlyy2060	January	China: Jilin	25.55 N 99.58 E	—	16S rRNA
W1706	*A. m. ligustica*	jlyy2060	January	China: Jilin	25.55 N 99.58 E	—	16S rRNA
W1792	*A. m. ligustica*	jlyy2061	January	China: Jilin	25.55 N 99.58 E	—	16S rRNA
W1794	*A. m. ligustica*	jlyy2061	January	China: Jilin	25.55 N 99.58 E	—	16S rRNA
W1796	*A. m. ligustica*	jlyy2061	January	China: Jilin	25.55 N 99.58 E	—	16S rRNA

W3872	*A. m. carnica*	sk11	March	China: Jilin	25.55 N 99.58 E	—	16S rRNA
W3874	*A. m. carnica*	sk11	March	China: Jilin	25.55 N 99.58 E	—	16S rRNA
W3876	*A. m. carnica*	sk11	March	China: Jilin	25.55 N 99.58 E	—	16S rRNA
W3956	*A. m. carnica*	sk12	March	China: Jilin	25.55 N 99.58 E	—	16S rRNA
W3958	*A. m. carnica*	sk12	March	China: Jilin	25.55 N 99.58 E	—	16S rRNA
W3960	*A. m. carnica*	sk12	March	China: Jilin	25.55 N 99.58 E	—	16S rRNA
W4212	*A. m. carnica*	jlsk3121	March	China: Jilin	25.55 N 99.58 E	—	16S rRNA
W4214	*A. m. carnica*	jlsk3121	March	China: Jilin	25.55 N 99.58 E	—	16S rRNA
W4216	*A. m. carnica*	jlsk3121	March	China: Jilin	25.55 N 99.58 E	—	16S rRNA
W4282	*A. m. carnica*	jlsk3028	March	China: Jilin	25.55 N 99.58 E	—	16S rRNA
W4284	*A. m. carnica*	jlsk3028	March	China: Jilin	25.55 N 99.58 E	—	16S rRNA
W4286	*A. m. carnica*	jlsk3028	March	China: Jilin	25.55 N 99.58 E	—	16S rRNA
W4366	*A. m. ligustica*	jlyy2060	March	China: Jilin	25.55 N 99.58 E	—	16S rRNA
W4368	*A. m. ligustica*	jlyy2060	March	China: Jilin	25.55 N 99.58 E	—	16S rRNA
W4370	*A. m. ligustica*	jlyy2060	March	China: Jilin	25.55 N 99.58 E	—	16S rRNA
W4414	*A. m. ligustica*	jlyy2061	March	China: Jilin	25.55 N 99.58 E	—	16S rRNA
W4416	*A. m. ligustica*	jlyy2061	March	China: Jilin	25.55 N 99.58 E	—	16S rRNA
W4418	*A. m. ligustica*	jlyy2061	March	China: Jilin	25.55 N 99.58 E	—	16S rRNA
W4488	*A. m. mellifera*	jloh5127	March	China: Jilin	25.55 N 99.58 E	—	16S rRNA

W4490	*A. m. mellifera*	jloh5127	March	China: Jilin	25.55 N 99.58 E	—	16S rRNA
W4492	*A. m. mellifera*	jloh5127	March	China: Jilin	25.55 N 99.58 E	—	16S rRNA
W4540	*A. m. mellifera*	jloh5140	March	China: Jilin	25.55 N 99.58 E	—	16S rRNA
W4542	*A. m. mellifera*	jloh5140	March	China: Jilin	25.55 N 99.58 E	—	16S rRNA
W4544	*A. m. mellifera*	jloh5140	March	China: Jilin	25.55 N 99.58 E	—	16S rRNA
W4894	*A. m. mellifera*	jloh5140	April	China: Jilin	25.55 N 99.58 E	—	16S rRNA
W4896	*A. m. mellifera*	jloh5140	April	China: Jilin	25.55 N 99.58 E	—	16S rRNA
W4898	*A. m. mellifera*	jloh5140	April	China: Jilin	25.55 N 99.58 E	—	16S rRNA
W4900	*A. m. mellifera*	jloh5140	April	China: Jilin	25.55 N 99.58 E	—	16S rRNA
W4902	*A. m. mellifera*	jloh5140	April	China: Jilin	25.55 N 99.58 E	—	16S rRNA
W4904	*A. m. mellifera*	jloh5140	April	China: Jilin	25.55 N 99.58 E	—	16S rRNA
W5002	*A. m. mellifera*	jloh5140	April	China: Jilin	25.55 N 99.58 E	—	16S rRNA
W5004	*A. m. mellifera*	jloh5140	April	China: Jilin	25.55 N 99.58 E	—	16S rRNA
W5006	*A. m. mellifera*	jloh5140	April	China: Jilin	25.55 N 99.58 E	—	16S rRNA
W5008	*A. m. mellifera*	jloh5140	April	China: Jilin	25.55 N 99.58 E	—	16S rRNA
W5010	*A. m. mellifera*	jloh5140	April	China: Jilin	25.55 N 99.58 E	—	16S rRNA
W5012	*A. m. mellifera*	jloh5140	April	China: Jilin	25.55 N 99.58 E	—	16S rRNA
W5158	*A. m. carnica*	jlsk3028	April	China: Jilin	25.55 N 99.58 E	—	16S rRNA
W5160	*A. m. carnica*	jlsk3028	April	China: Jilin	25.55 N 99.58 E	—	16S rRNA

W5162	*A. m. carnica*	jlsk3028	April	China: Jilin	25.55 N 99.58 E	—	16S rRNA
W5164	*A. m. carnica*	jlsk3028	April	China: Jilin	25.55 N 99.58 E	—	16S rRNA
W5166	*A. m. carnica*	jlsk3028	April	China: Jilin	25.55 N 99.58 E	—	16S rRNA
W5168	*A. m. carnica*	jlsk3028	April	China: Jilin	25.55 N 99.58 E	—	16S rRNA
W5210	*A. m. carnica*	jlsk3028	April	China: Jilin	25.55 N 99.58 E	—	16S rRNA
W5212	*A. m. carnica*	jlsk3028	April	China: Jilin	25.55 N 99.58 E	—	16S rRNA
W5214	*A. m. carnica*	jlsk3028	April	China: Jilin	25.55 N 99.58 E	—	16S rRNA
W5216	*A. m. carnica*	jlsk3028	April	China: Jilin	25.55 N 99.58 E	—	16S rRNA
W5218	*A. m. carnica*	jlsk3028	April	China: Jilin	25.55 N 99.58 E	—	16S rRNA
W5220	*A. m. carnica*	jlsk3028	April	China: Jilin	25.55 N 99.58 E	—	16S rRNA
W5328	*A. m. carnica*	jlsk3121	April	China: Jilin	25.55 N 99.58 E	—	16S rRNA
W5330	*A. m. carnica*	jlsk3121	April	China: Jilin	25.55 N 99.58 E	—	16S rRNA
W5332	*A. m. carnica*	jlsk3121	April	China: Jilin	25.55 N 99.58 E	—	16S rRNA
W5334	*A. m. carnica*	jlsk3121	April	China: Jilin	25.55 N 99.58 E	—	16S rRNA
W5336	*A. m. carnica*	jlsk3121	April	China: Jilin	25.55 N 99.58 E	—	16S rRNA
W5338	*A. m. carnica*	jlsk3121	April	China: Jilin	25.55 N 99.58 E	—	16S rRNA
W5402	*A. m. carnica*	jlsk3121	April	China: Jilin	25.55 N 99.58 E	—	16S rRNA
W5404	*A. m. carnica*	jlsk3121	April	China: Jilin	25.55 N 99.58 E	—	16S rRNA
W5406	*A. m. carnica*	jlsk3121	April	China: Jilin	25.55 N 99.58 E	—	16S rRNA

W5408	*A. m. carnica*	jlsk3121	April	China: Jilin	25.55 N 99.58 E	—	16S rRNA
W5410	*A. m. carnica*	jlsk3121	April	China: Jilin	25.55 N 99.58 E	—	16S rRNA
W5412	*A. m. carnica*	jlsk3121	April	China: Jilin	25.55 N 99.58 E	—	16S rRNA
W5578	*A. m. ligustica*	jlyy2060	April	China: Jilin	25.55 N 99.58 E	—	16S rRNA
W5580	*A. m. ligustica*	jlyy2060	April	China: Jilin	25.55 N 99.58 E	—	16S rRNA
W5582	*A. m. ligustica*	jlyy2060	April	China: Jilin	25.55 N 99.58 E	—	16S rRNA
W5584	*A. m. ligustica*	jlyy2060	April	China: Jilin	25.55 N 99.58 E	—	16S rRNA
W5586	*A. m. ligustica*	jlyy2060	April	China: Jilin	25.55 N 99.58 E	—	16S rRNA
W5588	*A. m. ligustica*	jlyy2060	April	China: Jilin	25.55 N 99.58 E	—	16S rRNA
W5628	*A. m. ligustica*	jlyy2060	April	China: Jilin	25.55 N 99.58 E	—	16S rRNA
W5630	*A. m. ligustica*	jlyy2060	April	China: Jilin	25.55 N 99.58 E	—	16S rRNA
W5632	*A. m. ligustica*	jlyy2060	April	China: Jilin	25.55 N 99.58 E	—	16S rRNA
W5634	*A. m. ligustica*	jlyy2060	April	China: Jilin	25.55 N 99.58 E	—	16S rRNA
W5636	*A. m. ligustica*	jlyy2060	April	China: Jilin	25.55 N 99.58 E	—	16S rRNA
W5638	*A. m. ligustica*	jlyy2060	April	China: Jilin	25.55 N 99.58 E	—	16S rRNA
W5890	*A. m. ligustica*	jlyy2061	April	China: Jilin	25.55 N 99.58 E	—	16S rRNA
W5892	*A. m. ligustica*	jlyy2061	April	China: Jilin	25.55 N 99.58 E	—	16S rRNA
W5894	*A. m. ligustica*	jlyy2061	April	China: Jilin	25.55 N 99.58 E	—	16S rRNA
W5896	*A. m. ligustica*	jlyy2061	April	China: Jilin	25.55 N 99.58 E	—	16S rRNA

W5898	*A. m. ligustica*	jlyy2061	April	China: Jilin	25.55 N 99.58 E	—	16S rRNA
W5900	*A. m. ligustica*	jlyy2061	April	China: Jilin	25.55 N 99.58 E	—	16S rRNA
W5942	*A. m. ligustica*	jlyy2061	April	China: Jilin	25.55 N 99.58 E	—	16S rRNA
W5944	*A. m. ligustica*	jlyy2061	April	China: Jilin	25.55 N 99.58 E	—	16S rRNA
W5946	*A. m. ligustica*	jlyy2061	April	China: Jilin	25.55 N 99.58 E	—	16S rRNA
W5948	*A. m. ligustica*	jlyy2061	April	China: Jilin	25.55 N 99.58 E	—	16S rRNA
W5950	*A. m. ligustica*	jlyy2061	April	China: Jilin	25.55 N 99.58 E	—	16S rRNA
W5952	*A. m. ligustica*	jlyy2061	April	China: Jilin	25.55 N 99.58 E	—	16S rRNA
W6106	*A. m. carnica*	sk11	April	China: Jilin	25.55 N 99.58 E	—	16S rRNA
W6110	*A. m. carnica*	sk11	April	China: Jilin	25.55 N 99.58 E	—	16S rRNA
W6112	*A. m. carnica*	sk11	April	China: Jilin	25.55 N 99.58 E	—	16S rRNA
W6114	*A. m. carnica*	sk11	April	China: Jilin	25.55 N 99.58 E	—	16S rRNA
W6116	*A. m. carnica*	sk11	April	China: Jilin	25.55 N 99.58 E	—	16S rRNA
W6118	*A. m. carnica*	sk11	April	China: Jilin	25.55 N 99.58 E	—	16S rRNA
W6160	*A. m. carnica*	sk12	April	China: Jilin	25.55 N 99.58 E	—	16S rRNA
W6162	*A. m. carnica*	sk12	April	China: Jilin	25.55 N 99.58 E	—	16S rRNA
W6164	*A. m. carnica*	sk12	April	China: Jilin	25.55 N 99.58 E	—	16S rRNA
W6166	*A. m. carnica*	sk12	April	China: Jilin	25.55 N 99.58 E	—	16S rRNA
W6168	*A. m. carnica*	sk12	April	China: Jilin	25.55 N 99.58 E	—	16S rRNA

W6170	*A. m. carnica*	sk12	April	China: Jilin	25.55 N 99.58 E	—	16S rRNA
B11502	*A. m. carnica*	jlsk3121	Jun	China: Jilin	25.55 N 99.58 E		16S rRNA
B11503	*A. m. carnica*	jlsk3121	Jun	China: Jilin	25.55 N 99.58 E		16S rRNA
B11504	*A. m. carnica*	jlsk3121	Jun	China: Jilin	25.55 N 99.58 E		16S rRNA
B11505	*A. m. carnica*	jlsk3121	Jun	China: Jilin	25.55 N 99.58 E		16S rRNA
B11506	*A. m. carnica*	jlsk3121	Jun	China: Jilin	25.55 N 99.58 E		16S rRNA
B11551	*A. m. carnica*	jlsk3028	Jun	China: Jilin	25.55 N 99.58 E		16S rRNA
B11552	*A. m. carnica*	jlsk3028	Jun	China: Jilin	25.55 N 99.58 E		16S rRNA
B11553	*A. m. carnica*	jlsk3028	Jun	China: Jilin	25.55 N 99.58 E		16S rRNA
B11555	*A. m. carnica*	jlsk3028	Jun	China: Jilin	25.55 N 99.58 E		16S rRNA
B11556	*A. m. carnica*	jlsk3028	Jun	China: Jilin	25.55 N 99.58 E		16S rRNA
B9831	*A. m. mellifera*	jloh5140	Jun	China: Jilin	25.55 N 99.58 E		16S rRNA
B9832	*A. m. mellifera*	jloh5140	Jun	China: Jilin	25.55 N 99.58 E		16S rRNA
B9834	*A. m. mellifera*	jloh5140	Jun	China: Jilin	25.55 N 99.58 E		16S rRNA
B9835	*A. m. mellifera*	jloh5140	Jun	China: Jilin	25.55 N 99.58 E		16S rRNA
B9836	*A. m. mellifera*	jloh5140	Jun	China: Jilin	25.55 N 99.58 E		16S rRNA
B9883	*A. m. mellifera*	jlyy2061	Jun	China: Jilin	25.55 N 99.58 E		16S rRNA
B9884	*A. m. ligustica*	jlyy2061	Jun	China: Jilin	25.55 N 99.58 E		16S rRNA
B9885	*A. m. ligustica*	jlyy2061	Jun	China: Jilin	25.55 N 99.58 E		16S rRNA

B9886	*A. m. ligustica*	jlyy2061	Jun	China: Jilin	25.55 N 99.58 E		16S rRNA
B9887	*A. m. ligustica*	jlyy2061	Jun	China: Jilin	25.55 N 99.58 E		16S rRNA
B9932	*A. m. ligustica*	jlyy2060	Jun	China: Jilin	25.55 N 99.58 E		16S rRNA
B9934	*A. m. ligustica*	jlyy2060	Jun	China: Jilin	25.55 N 99.58 E		16S rRNA
B9935	*A. m. ligustica*	jlyy2060	Jun	China: Jilin	25.55 N 99.58 E		16S rRNA
B9936	*A. m. ligustica*	jlyy2060	Jun	China: Jilin	25.55 N 99.58 E		16S rRNA
B9937	*A. m. ligustica*	jlyy2060	Jun	China: Jilin	25.55 N 99.58 E		16S rRNA
B11501	*A. m. carnica*	jlsk3028	Jun	China: Jilin	25.55 N 99.58 E	metagenome	—
B11504	*A. m. carnica*	jlsk3028	Jun	China: Jilin	25.55 N 99.58 E	metagenome	—
B11507	*A. m. carnica*	jlsk3028	Jun	China: Jilin	25.55 N 99.58 E	metagenome	—
B11508	*A. m. carnica*	jlsk3028	Jun	China: Jilin	25.55 N 99.58 E	metagenome	—
B11509	*A. m. carnica*	jlsk3028	Jun	China: Jilin	25.55 N 99.58 E	metagenome	—
B11510	*A. m. carnica*	jlsk3028	Jun	China: Jilin	25.55 N 99.58 E	metagenome	—
B11554	*A. m. carnica*	jlsk3121	Jun	China: Jilin	25.55 N 99.58 E	metagenome	—
B11555	*A. m. carnica*	jlsk3121	Jun	China: Jilin	25.55 N 99.58 E	metagenome	—
B11557	*A. m. carnica*	jlsk3121	Jun	China: Jilin	25.55 N 99.58 E	metagenome	—
B11558	*A. m. carnica*	jlsk3121	Jun	China: Jilin	25.55 N 99.58 E	metagenome	—
B11559	*A. m. carnica*	jlsk3121	Jun	China: Jilin	25.55 N 99.58 E	metagenome	—
B11560	*A. m. carnica*	jlsk3121	Jun	China: Jilin	25.55 N 99.58 E	metagenome	—

B9832	*A. m. mellifera*	jloh5140	Jun	China: Jilin	25.55 N 99.58 E	metagenome	—
B9833	*A. m. mellifera*	jloh5140	Jun	China: Jilin	25.55 N 99.58 E	metagenome	—
B9838	*A. m. mellifera*	jloh5140	Jun	China: Jilin	25.55 N 99.58 E	metagenome	—
B9839	*A. m. mellifera*	jloh5140	Jun	China: Jilin	25.55 N 99.58 E	metagenome	—
B9840	*A. m. mellifera*	jloh5140	Jun	China: Jilin	25.55 N 99.58 E	metagenome	—
B9841	*A. m. mellifera*	jloh5140	Jun	China: Jilin	25.55 N 99.58 E	metagenome	—
B9842	*A. m. mellifera*	jloh5140	Jun	China: Jilin	25.55 N 99.58 E	metagenome	—
B9843	*A. m. mellifera*	jloh5140	Jun	China: Jilin	25.55 N 99.58 E	metagenome	—
B9846	*A. m. mellifera*	jloh5140	Jun	China: Jilin	25.55 N 99.58 E	metagenome	—
B9848	*A. m. mellifera*	jloh5140	Jun	China: Jilin	25.55 N 99.58 E	metagenome	—
B9849	*A. m. mellifera*	jloh5140	Jun	China: Jilin	25.55 N 99.58 E	metagenome	—
B9850	*A. m. mellifera*	jloh5140	Jun	China: Jilin	25.55 N 99.58 E	metagenome	—
B9851	*A. m. mellifera*	jloh5140	Jun	China: Jilin	25.55 N 99.58 E	metagenome	—
B9852	*A. m. mellifera*	jloh5140	Jun	China: Jilin	25.55 N 99.58 E	metagenome	—
B9854	*A. m. mellifera*	jloh5140	Jun	China: Jilin	25.55 N 99.58 E	metagenome	—
B9855	*A. m. mellifera*	jloh5140	Jun	China: Jilin	25.55 N 99.58 E	metagenome	—
B9856	*A. m. mellifera*	jloh5140	Jun	China: Jilin	25.55 N 99.58 E	metagenome	—
B9857	*A. m. mellifera*	jloh5140	Jun	China: Jilin	25.55 N 99.58 E	metagenome	—
B9858	*A. m. mellifera*	jloh5140	Jun	China: Jilin	25.55 N 99.58 E	metagenome	—

B9859	*A. m. mellifera*	jloh5140	Jun	China: Jilin	25.55 N 99.58 E	metagenome	—
B9860	*A. m. mellifera*	jloh5140	Jun	China: Jilin	25.55 N 99.58 E	metagenome	—
B9861	*A. m. mellifera*	jloh5140	Jun	China: Jilin	25.55 N 99.58 E	metagenome	—
B9862	*A. m. mellifera*	jloh5140	Jun	China: Jilin	25.55 N 99.58 E	metagenome	—
B9863	*A. m. mellifera*	jloh5140	Jun	China: Jilin	25.55 N 99.58 E	metagenome	—
B9864	*A. m. mellifera*	jloh5140	Jun	China: Jilin	25.55 N 99.58 E	metagenome	—
B9865	*A. m. mellifera*	jloh5140	Jun	China: Jilin	25.55 N 99.58 E	metagenome	—
B9866	*A. m. mellifera*	jloh5140	Jun	China: Jilin	25.55 N 99.58 E	metagenome	—
B9867	*A. m. mellifera*	jloh5140	Jun	China: Jilin	25.55 N 99.58 E	metagenome	—
B9868	*A. m. mellifera*	jloh5140	Jun	China: Jilin	25.55 N 99.58 E	metagenome	—
B9869	*A. m. mellifera*	jloh5140	Jun	China: Jilin	25.55 N 99.58 E	metagenome	—
B9870	*A. m. mellifera*	jloh5140	Jun	China: Jilin	25.55 N 99.58 E	metagenome	—
B9871	*A. m. mellifera*	jloh5140	Jun	China: Jilin	25.55 N 99.58 E	metagenome	—
B9881	*A. m. ligustica*	jlyy2060	Jun	China: Jilin	25.55 N 99.58 E	metagenome	—
B9883	*A. m. ligustica*	jlyy2060	Jun	China: Jilin	25.55 N 99.58 E	metagenome	—
B9889	*A. m. ligustica*	jlyy2060	Jun	China: Jilin	25.55 N 99.58 E	metagenome	—
B9890	*A. m. ligustica*	jlyy2060	Jun	China: Jilin	25.55 N 99.58 E	metagenome	—
B9891	*A. m. ligustica*	jlyy2060	Jun	China: Jilin	25.55 N 99.58 E	metagenome	—
B9892	*A. m. ligustica*	jlyy2060	Jun	China: Jilin	25.55 N 99.58 E	metagenome	—

B9933	*A. m. ligustica*	jlyy2061	Jun	China: Jilin	25.55 N 99.58 E	metagenome	—
B9938	*A. m. ligustica*	jlyy2061	Jun	China: Jilin	25.55 N 99.58 E	metagenome	—
B9939	*A. m. ligustica*	jlyy2061	Jun	China: Jilin	25.55 N 99.58 E	metagenome	—
B9940	*A. m. ligustica*	jlyy2061	Jun	China: Jilin	25.55 N 99.58 E	metagenome	—
B9941	*A. m. ligustica*	jlyy2061	Jun	China: Jilin	25.55 N 99.58 E	metagenome	—
B9942	*A. m. ligustica*	jlyy2061	Jun	China: Jilin	25.55 N 99.58 E	metagenome	—
