# Supplementary material for: Community Dynamics in Structure and Function of Honey Bee Gut Bacteria in Response to Winter Dietary Shift
Source: mBio. 2022 Aug 29;13(5):e01131-22. doi: 10.1128/mbio.01131-22 (PMC9600256; doi:10.1128/mbio.01131-22)
Supplement: TABLE S2 [file mbio.01131-22-s0002.pdf]

		June			November			January			March		
**pathway**	**compound_name**	**B11524**	**B11525**	**B11526**	**W6950**	**W6952**	**W6954**	**W2042**	**W2044**	**W2046**	**W3394**	**W3396**	**W3398**
**Pollen metabolites**	Keracyanin	9383933.9	3804478.3	7073544.3	92310.394	55939.531	35393.669	60400	58231.497	56090.951	44289.477	55719.585	66780.101
	Kaempferol	31036411	47169921	25649584	459667.31	159303.4	119730.59	122039.81	74903.677	81424.459	265134.13	724292.07	72418.332
	Quercitrin	268449.04	494597.75	529423.23	75809.489	78162.079	36807.284	49840.969	75088.533	46421.062	53854.663	57590.491	61809.068
	9,10-Dihydroxystearic acid	45482913	52795333	60043235	4620712.5	8013367.4	62291193	4009416.9	4643405.5	17343736	5821511.6	9043565.2	11505611
	tricoumaroyl spermidine	1414281.7	4802312.1	10196465	42680.364	35305.869	70090.342	35102.929	45013.667	33054.798	32569.901	43525.368	45519.846
	Spermidine	54178243	44233079	50858113	45459894	15072186	22054417	26879577	30738408	9766931.9	6194040.1	4645456.6	14805750
**Phenylalanine, tyrosine and tryptophan biosynthesis**	(-)-Chorismic acid	65333.612	50286.849	56706.895	92022.889	85868.916	76064.709	86929.831	76429.281	139611.46	75721.52	147739	109962.47
	4-Hydroxyphenylpyruvic acid	430780.65	911216.65	257854.91	50754.159	62671.571	53009.58	43297.998	41376.837	48087.502	45013.481	31922.058	131216.85
	D-(-)-Quinic acid	437154.32	324416.47	460784.54	1605521.4	1257788.6	2381041.2	2003594.6	2600524.4	4701429.3	4728539.7	4466573.9	1400894.7
	D-Erythrose 4-phosphate	596106.82	566219.14	712119.87	1427825.7	1113771.9	1026391.1	1016987.6	836950.03	978728.78	802704.03	531305.83	876918.69
	D-Sedoheptulose 7-phosphate	299754.5	374221.82	535451.29	676303.15	994674.92	673173.15	312056.02	200486.76	202428.17	191813.32	69219.396	86437.975
	Shikimic Acid	292572.84	249028.25	280791.52	901332.84	454443.66	2006086	1347251.6	1973915.6	3887817.9	1776552.7	1540235.1	1016708.3
	pretyrosine	950072.22	1124759.5	1621962.2	1790770.1	640060.46	870993.56	1442299.4	1756713.2	1366364.4	3115226.1	2539968	3025826.6
**Tryptophan metabolism**	5-Hydroxy-N-formylkynurenine	9704.1072	6140.0005	8179.6158	11646.85	11005.106	6411.2329	12304.912	11154.808	12643.247	23371.235	24672.989	21576.764
	Formylkynurenine	10012821	1992077.9	3865793.2	2912562.3	300712.73	163250.56	493768.2	96485.931	108314.48	506344.95	738836.4	120999.04
	L-3-hydroxykynurenine	522985.02	676804.33	994483.01	346871.87	102220.79	419418.37	313315.89	798055.18	419379.44	1111268.8	1248413	2743819.4
	L-Kynurenine	2350773.1	4546097.7	2683833.2	17515035	832511.01	266762.21	237771.43	252324.44	226051.95	537352.51	1896035.6	366893.49
	Tryptamine	86925.321	134155.64	59895.239	131737.46	50072.484	321180.65	725470.97	304639.33	143146.86	253028.94	299375.83	142817.54
	5-Hydroxytryptophan	358518.85	209140.62	212705.82	771274.19	301715.17	429863.32	226235.56	269433.32	250438.19	193746.12	309155.59	143573.97
	Serotonin	52034.496	236664.4	40450.159	130294.94	85914.072	125278.87	82035.849	64164.444	91635.43	42016.11	149437.5	22839.477
**Tyrosine metabolism**	L-Dopa	5424380.1	3731558.5	3328505.7	1717854.5	1918210.6	1193660.3	972391.29	906760.72	1452567	2576968	1474691	855212.46
	Tyramine	548271.67	913730.7	496814.7	944741.71	573373.49	2687968.1	2680948.8	2482720.4	1814530.7	1437553.7	5310461.9	710198.44
