# Supplementary figures and images for: Community Dynamics in Structure and Function of Honey Bee Gut Bacteria in Response to Winter Dietary Shift
Source: mBio. 2022 Aug 29;13(5):e01131-22. doi: 10.1128/mbio.01131-22 (PMC9600256; doi:10.1128/mbio.01131-22)

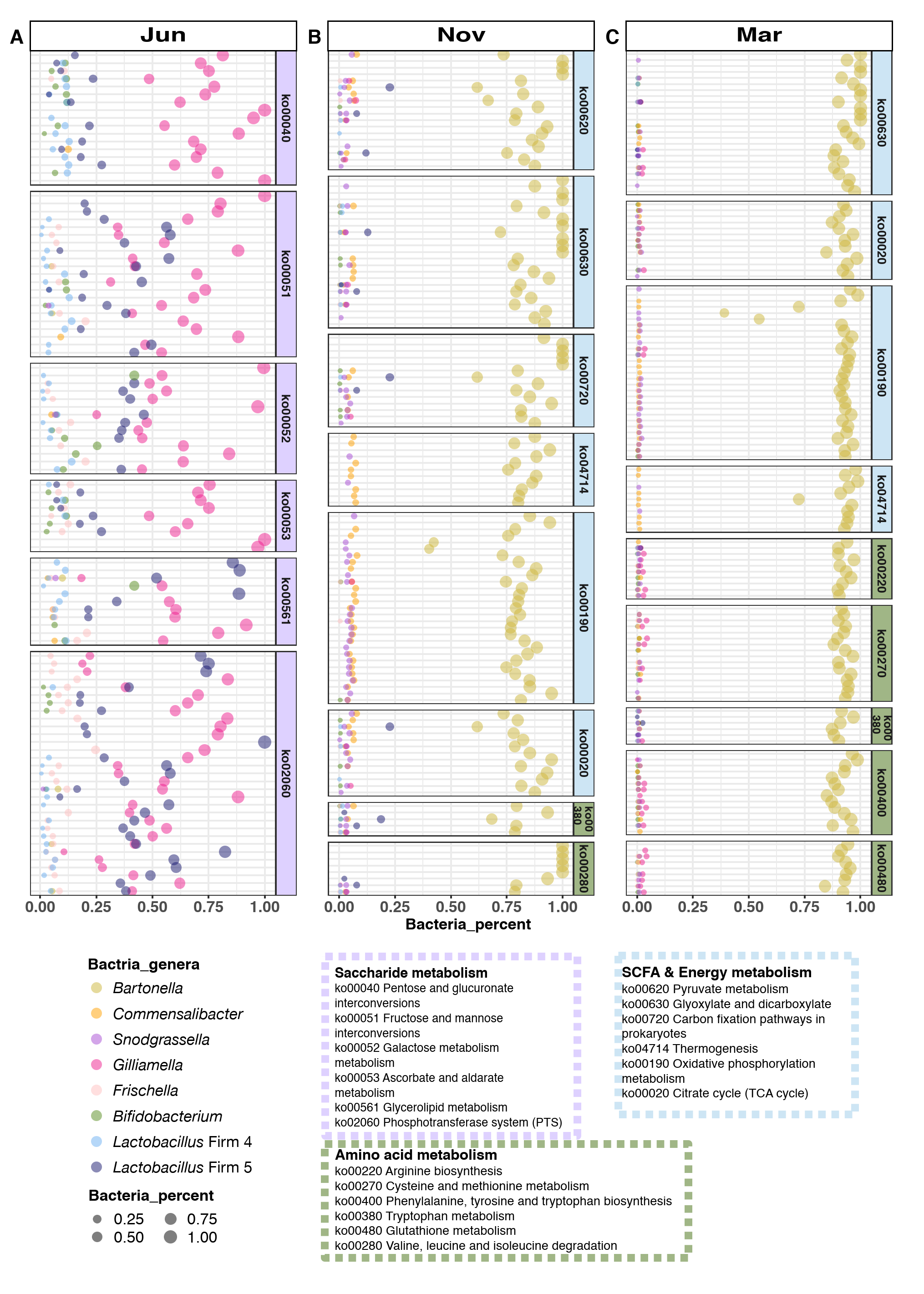

Supplement: FIG S3 [file mbio.01131-22-s0008.tif]
