# Supplementary material for: Community Dynamics in Structure and Function of Honey Bee Gut Bacteria in Response to Winter Dietary Shift
Source: mBio. 2022 Aug 29;13(5):e01131-22. doi: 10.1128/mbio.01131-22 (PMC9600256; doi:10.1128/mbio.01131-22)
Supplement: TABLE S3 [file mbio.01131-22-s0003.pdf]

Pathway	Phenylalanine tyrosine and tryptophan biosynthesis												Phenylalanine and tyrosine biosynthesis									Tryptophan biosynthesis											General L-amino acid transport system substrate-binding protein			
EC	EC: 1.1.1.25	EC: 2.5.1.19	EC: 2.7.1.71	EC: 2.5.1.54	EC: 4.2.3.4	EC: 4.2.3.5	EC: 4.2.1.10	EC: 4.2.1.10	EC: 1.1.5.8	EC: 2.7.1.71; 4.2.3.4	EC: 5.4.99.5; 4.2.1.51	EC: 5.4.99.5; 1.3.1.12	EC: 1.3.1.43; 1.3.1.12	EC: 2.6.1.1	EC: 2.6.1.1	EC: 2.6.1.9	EC: 2.6.1.57	EC: 4.2.1.51; 4.2.1.91	EC: 5.4.99.5	EC: 1.3.1.12	EC: 4.2.1.51	EC: 2.4.2.18	EC: 4.1.1.48	EC: 4.1.3.27	EC: 4.1.3.27	EC: 4.2.1.20	EC: 4.2.1.20	EC: 5.3.1.24	EC: 4.1.3.27; 2.4.2.18	EC: 4.1.1.48; 5.3.1.24	EC: 4.1.3.27	EC: 5.3.1.16; 5.3.1.24				
KO	K00014	K00800	K00891	K01626	K01735	K01736	K03785	K03786	K05358	K13829	K14170	K14187	K00220	K00812	K00813	K00817	K00832	K01713	K04092	K04517	K04518	K00766	K01609	K01657	K01658	K01695	K01696	K01817	K13497	K13498	K13503	K24017	K09969	K09970	K09971	K09972
Bifidobacterium sp 7101	1	1	1	2	1	1	0	1	0	1	0	0	0	1	0	2	0	0	1	1	0	1	1	0	0	1	1	0	0	0	0	1	0	0	0	0
Gilliamella apicola A-1-24	4	1	3	1	1	1	2	0	0	0	1	1	0	0	1	1	1	1	0	0	1	1	1	1	1	1	1	0	1	1	0	0	0	0	0	0
Gilliamella apicola A-12-12	3	1	2	1	1	1	1	0	0	0	1	1	0	0	1	1	1	1	0	0	1	1	1	1	1	1	1	0	1	1	0	0	0	0	0	0
Gilliamella apicola A-2-24	4	1	3	1	1	1	2	0	0	0	1	1	0	0	1	1	1	1	0	0	1	1	1	1	1	1	1	0	1	1	0	0	0	0	0	0
Gilliamella apis A-4-12	3	1	2	1	1	1	1	0	0	0	1	1	0	0	1	1	1	0	0	0	1	1	1	1	0	1	1	0	1	1	0	0	0	0	0	0
Gilliamella apicola A-7-12	4	1	3	1	1	1	2	0	0	0	1	1	0	0	1	1	1	1	0	0	1	1	1	1	1	1	1	0	1	1	0	0	0	0	0	0
Gilliamella apicola A-7-24	3	1	2	1	1	1	1	0	0	0	1	1	0	0	1	1	1	1	0	0	1	1	1	1	1	1	1	0	1	1	0	0	0	0	0	0
Gilliamella apicola A-8-12	3	1	2	1	1	1	1	0	0	0	1	1	0	0	1	1	1	1	0	0	1	1	1	1	1	1	1	0	1	1	0	0	0	0	0	0
Gilliamella apicola A-9-12	4	1	3	1	1	1	2	0	0	0	1	1	0	0	1	1	1	1	0	0	1	1	1	1	1	1	1	0	1	1	0	0	0	0	0	0
Snodgrassella alvi A-9-24	1	1	1	1	1	1	0	1	1	0	1	0	0	0	0	1	1	0	0	1	1	1	1	1	1	1	1	1	0	0	0	0	0	0	0	0
Gilliamella apis A-TSA1	3	1	2	1	1	1	1	0	0	0	1	1	0	0	1	1	1	0	0	0	1	1	0	1	1	1	1	0	1	1	0	0	0	0	0	0
Gilliamella apis A-TSA2	3	1	2	1	1	1	1	0	0	0	1	1	0	0	1	1	1	0	0	0	1	1	0	1	1	1	1	0	1	1	0	0	0	0	0	0
Gilliamella apis A-TSA3	3	1	2	1	1	1	1	0	0	0	1	1	0	0	1	1	1	0	0	0	1	1	0	1	1	1	1	0	1	1	0	0	0	0	0	0
Gilliamella apis A-TSA4	3	1	2	1	1	1	1	0	0	0	1	1	0	0	1	1	1	0	0	0	1	1	0	1	1	1	1	0	1	1	0	0	0	0	0	0
Bifidobacterium sp A11	1	1	1	2	1	1	0	1	0	1	0	0	0	1	0	2	0	0	1	1	0	1	1	0	0	1	1	0	0	0	0	1	0	0	0	0
Snodgrassella alvi A12	1	1	1	1	1	1	0	1	1	0	1	0	0	0	0	1	1	0	0	1	1	1	1	1	1	1	1	1	0	0	0	0	0	0	0	0
Snodgrassella alvi A5	1	1	1	1	1	1	0	1	1	0	1	0	0	0	0	1	1	0	0	1	1	1	1	1	1	1	1	1	0	0	0	0	0	0	0	0
Gilliamella apicola A8	4	1	3	1	1	1	2	0	0	0	1	1	0	0	1	1	1	1	0	0	1	1	1	1	1	1	1	0	1	1	0	0	0	0	0	0
Gilliamella apicola A9	4	1	3	1	1	1	2	0	0	0	1	1	0	0	1	1	1	1	0	0	1	1	1	1	1	1	1	0	1	1	0	0	0	0	0	0
Gilliamella apis AM1	3	1	2	1	1	1	1	0	0	0	1	1	0	0	1	1	1	0	0	0	1	1	0	1	1	1	1	0	1	1	0	0	0	0	0	0
Gilliamella apicola AM4	4	1	3	1	1	1	2	0	0	0	1	1	0	0	1	1	1	1	0	0	1	1	1	1	1	1	1	1	1	1	0	0	0	0	0	0
Gilliamella apicola AM6	4	1	3	1	1	1	2	0	0	0	1	1	0	0	1	1	1	1	0	0	1	1	1	1	1	1	1	1	1	1	0	0	0	0	0	0
Gilliamella apicola Aw-17	3	1	2	1	1	1	1	0	0	0	1	1	0	0	1	1	1	1	0	0	1	1	1	1	1	1	1	0	1	1	0	0	0	0	0	0
Gilliamella apicola AW11	3	1	2	1	1	1	1	0	0	0	1	1	0	0	1	1	1	1	0	0	1	1	1	1	1	1	1	0	1	1	0	0	0	0	0	0
Gilliamella apicola AW13	4	1	3	1	1	1	2	0	0	0	1	1	0	0	1	1	1	1	0	0	1	1	1	1	1	1	1	0	1	1	0	0	0	0	0	0
Bartonella apis BBC0122	2	1	1	1	1	1	0	1	2	0	0	0	1	1	0	2	0	1	1	0	1	1	1	0	0	1	1	1	0	0	1	0	1	1	1	1
Bartonella apis BBC0178	1	1	1	1	1	1	0	1	2	0	0	0	1	1	0	2	0	1	1	0	1	1	1	0	0	1	1	1	0	0	1	0	1	1	1	1
Bartonella apis BBC0244	1	1	1	1	1	1	0	1	2	0	0	0	1	2	0	2	0	1	1	0	1	1	1	0	0	1	1	1	0	0	1	0	1	1	1	2
Bartonella apis PEB0122	1	1	0	1	1	1	0	1	2	0	0	0	1	1	0	2	0	1	1	0	1	1	1	0	0	1	1	1	0	0	1	0	1	1	1	1
Bartonella apis PEB0149	1	1	0	1	1	1	0	1	2	0	0	0	1	1	0	2	0	1	1	0	1	1	1	0	0	1	1	1	0	0	1	0	1	1	1	1
Bartonella apis PEB0150	2	1	0	1	1	1	0	1	2	0	0	0	1	1	0	2	0	1	1	0	1	1	1	0	0	1	1	1	0	0	1	0	1	1	1	1
Bartonella apis W7133	1	1	1	1	1	1	0	1	2	0	0	0	1	1	0	2	0	1	1	0	1	1	1	0	0	1	1	1	0	0	1	0	1	1	1	1
Lactobacillus kullabergensis Biut2	2	0	0	0	0	0	1	0	0	0	0	0	0	0	0	0	0	0	0	0	0	0	0	0	0	0	0	0	0	0	0	0	0	0	0	0
Lactobacillus helsingborgensis Bma5	1	0	0	0	0	0	1	0	0	0	0	0	0	0	0	0	0	0	0	0	0	0	0	0	0	0	0	0	0	0	0	0	0	0	0	0
Bifidobacterium coryneforme Bma6	1	1	1	2	1	1	0	1	0	1	0	0	0	1	0	2	0	0	1	1	0	1	1	0	0	1	1	0	0	0	0	1	0	0	0	0
Bifidobacterium asteroides DSM-20089	1	1	1	2	1	1	0	1	0	1	0	0	0	1	0	2	0	0	1	1	0	1	1	0	0	1	1	0	0	0	0	1	0	0	0	0
Bifidobacterium coryneforme DSM-20216	1	1	1	2	1	1	0	1	0	1	0	0	0	1	0	2	0	0	1	1	0	1	1	0	0	1	1	0	0	0	0	1	0	0	0	0
Gilliamella apis ESL0169	3	1	2	1	1	1	1	0	0	0	1	1	0	0	1	1	1	0	0	0	1	1	0	1	0	1	1	0	1	1	0	0	0	0	0	0
Bifidobacterium asteroides ESL0170	1	1	1	2	1	1	0	1	0	1	0	0	0	1	0	2	0	0	1	1	0	1	1	0	0	1	1	0	0	0	0	1	0	0	0	0
Gilliamella apis ESL0172	3	1	2	1	1	1	1	0	0	0	1	1	0	0	1	1	1	0	0	0	1	1	0	1	0	1	1	0	1	1	0	0	0	0	0	0
Gilliamella apicola ESL0177	3	1	3	1	1	1	1	0	0	0	1	1	0	0	1	1	1	0	0	0	1	1	0	1	1	1	1	0	1	1	0	0	0	0	0	0
Gilliamella apicola ESL0178	4	1	3	1	1	1	2	0	0	0	1	1	0	0	1	1	1	1	0	0	1	1	1	1	1	1	1	0	1	1	0	0	0	0	0	0
Gilliamella apicola ESL0182	3	1	3	1	1	1	1	0	0	0	1	1	0	0	1	1	1	0	0	0	1	1	1	1	1	1	1	0	1	1	0	0	0	0	0	0
Lactobacillus melliventris ESL0184	1	0	0	0	0	0	1	0	0	0	0	0	0	0	0	0	0	0	0	0	0	0	0	0	0	0	0	0	0	0	0	0	0	0	0	0
Snodgrassella alvi ESL0196	1	1	1	1	1	1	0	1	1	0	1	0	0	0	0	1	1	0	0	1	1	1	1	1	1	1	1	1	0	0	0	0	0	0	0	0
Bifidobacterium indicum ESL0197	1	1	1	2	1	1	0	1	0	1	0	0	0	1	0	2	0	0	1	1	0	1	1	0	0	1	1	0	0	0	0	1	0	0	0	0
Bifidobacterium asteroides ESL0198	1	1	1	2	1	1	0	1	0	1	0	0	0	1	0	2	0	0	1	1	0	1	1	0	0	1	1	0	0	0	0	1	0	0	0	0
Bifidobacterium asteroides ESL0199	1	1	1	2	1	1	0	1	0	1	0	0	0	1	0	2	0	0	1	1	0	1	1	0	0	1	1	0	0	0	0	1	0	0	0	0
Bifidobacterium asteroides ESL0200	1	1	1	2	1	1	0	1	0	1	0	0	0	1	0	2	0	0	1	1	0	1	0	0	0	1	1	0	0	0	0	1	0	0	0	0
Lactobacillus sp ESL0260	1	0	0	0	0	0	1	0	0	0	0	0	0	0	0	0	0	0	0	0	0	0	0	0	0	0	0	0	0	0	0	0	0	0	0	0
Lactobacillus sp ESL0261	1	0	0	0	0	0	1	0	0	0	0	0	0	0	0	0	0	0	0	0	0	0	0	0	0	0	0	0	0	0	0	0	0	0	0	0
Lactobacillus sp ESL0262	1	0	0	0	0	0	1	0	0	0	0	0	0	0	0	0	0	0	0	0	0	0	0	0	0	0	0	0	0	0	0	0	0	0	0	0
Lactobacillus kimbladii Hma2	2	0	0	0	0	0	1	0	0	0	0	0	0	0	0	0	0	0	0	0	0	0	0	0	0	0	0	0	0	0	0	0	0	0	0	0
Bifidobacterium asteroides Hma3	1	1	1	2	1	1	0	1	0	1	0	0	0	1	0	2	0	0	1	1	0	1	1	0	0	1	1	0	0	0	0	1	0	0	0	0
Lactobacillus melliventris Hma8	1	0	0	0	0	0	1	0	0	0	0	0	0	0	0	0	0	0	0	0	0	0	0	0	0	0	0	0	0	0	0	0	0	0	0	0
Lactobacillus mellis Hon2	0	1	1	0	0	1	0	0	0	0	0	0	0	0	0	0	0	0	0	1	0	0	0	0	0	0	0	0	0	0	0	0	0	0	0	0
Bifidobacterium indicum LMG 11587	1	1	1	2	1	1	0	1	0	1	0	0	0	1	0	2	0	0	1	1	0	1	1	0	0	1	1	0	0	0	0	1	0	0	0	0
Gilliamella apis M1-2G	3	1	2	1	1	1	1	0	0	0	1	1	0	0	1	1	1	0	0	0	1	1	0	1	0	1	1	0	1	1	0	0	0	0	0	0
Gilliamella apicola M6-3G	3	1	2	1	1	1	1	0	0	0	1	1	0	0	1	1	1	0	0	0	1	1	0	1	0	1	1	0	1	1	0	0	0	0	0	0
Gilliamella apicola N-12-12	4	1	3	1	1	1	2	0	0	0	1	1	0	0	1	1	1	1	0	0	1	1	1	1	1	1	1	0	1	1	0	0	0	0	0	0
Gilliamella apicola N-15-12	4	1	3	1	1	1	2	0	0	0	1	1	0	0	1	1	1	1	0	0	1	1	1	1	1	1	1	0	1	1	0	0	0	0	0	0
Gilliamella apicola N-22	4	1	3	1	1	1	2	0	0	0	1	1	0	0	1	1	1	1	0	0	1	1	1	1	1	1	1	0	1	1	0	0	0	0	0	0
Gilliamella apis N-G1	3	1	2	1	1	1	1	0	0	0	1	1	0	0	1	1	1	0	0	0	1	1	0	1	0	1	1	0	1	1	0	0	0	0	0	0
Gilliamella sp N-G2	3	1	2	1	1	1	1	0	1	0	1	1	0	0	1	1	1	0	0	0	1	1	0	1	0	1	1	0	1	1	0	0	0	0	0	0
Gilliamella apis N-G3	3	1	2	1	1	1	1	0	0	0	1	1	0	0	1	1	1	0	0	0	1	1	0	1	0	1	1	0	1	1	0	0	0	0	0	0
Gilliamella apis N-G4	3	1	2	1	1	1	1	0	0	0	1	1	0	0	1	1	1	0	0	0	1	1	0	1	0	1	1	0	1	1	0	0	0	0	0	0
Gilliamella apicola N-G5	4	1	3	1	1	1	2	0	0	0	1	1	0	0	1	1	1	1	0	0	1	1	1	1	1	1	1	0	1	1	0	0	0	0	0	0
Snodgrassella alvi N-S2	1	1	1	1	1	1	0	1	1	0	1	0	0	0	0	1	1	0	0	1	1	1	1	1	1	1	1	1	0	0	0	0	0	0	0	0
Snodgrassella alvi N-S4	1	1	1	1	1	1	0	1	1	0	1	0	0	0	0	1	1	0	0	1	1	1	1	1	1	1	1	1	0	0	0	0	0	0	0	0
Gilliamella sp N-W3	3	1	2	1	1	1	1	0	1	0	1	1	0	0	1	1	1	0	0	0	1	1	0	1	0	1	1	0	1	1	0	0	0	0	0	0
Snodgrassella alvi N-W7	1	1	1	1	1	1	0	1	1	0	1	0	0	0	0	1	1	0	0	1	1	1	1	1	1	1	1	1	0	0	0	0	0	0	0	0
Gilliamella apicola N10	3	1	2	1	1	1	1	0	0	0	1	1	0	0	1	1	1	1	0	0	1	1	1	1	1	1	1	0	1	1	0	0	0	0	0	0
Gilliamella apicola N2	3	1	2	1	1	1	1	0	0	0	1	1	0	0	1	1	1	1	0	0	1	1	1	1	1	1	1	0	1	1	0	0	0	0	0	0
Gilliamella apicola N4	3	1	2	1	1	1	1	0	0	0	1	1	0	0	1	1	1	1	0	0	1	1	1	1	1	1	1	0	1	1	0	0	0	0	0	0
Gilliamella apicola N6	3	1	2	1	1	1	1	0	0	0	1	1	0	0	1	1	1	1	0	0	1	1	1	1	1	1	1	0	1	1	0	0	0	0	0	0
Gilliamella apis NO1	3	1	2	1	1	1	1	0	0	0	1	1	0	0	1	1	1	0	0	0	1	1	1	1	0	1	1	0	1	1	0	0	0	0	0	0
Gilliamella apicola NO10	4	1	3	1	1	1	2	0	0	0	1	1	0	0	1	1	1	1	0	0	1	1	1	1	1	1	1	0	1	1	0	0	0	0	0	0
Gilliamella apis NO12	3	1	2	1	1	1	1	0	0	0	1	1	0	0	1	1	1	0	0	0	1	1	1	1	0	1	1	0	1	1	0	0	0	0	0	0
Gilliamella apis NO13	3	1	2	1	1	1	1	0	0	0	1	1	0	0	1	1	1	0	0	0	1	1	1	1	0	1	1	0	1	1	0	0	0	0	0	0
Gilliamella apis NO14	3	1	2	1	1	1	1	0	0	0	1	1	0	0	1	1	1	0	0	0	1	1	1	1	0	1	1	0	1	1	0	0	0	0	0	0
Gilliamella apis NO15	3	1	2	1	1	1	1	0	0	0	1	1	0	0	1	1	1	0	0	0	1	1	1	1	0	1	1	0	1	1	0	0	0	0	0	0
Gilliamella apis NO16	3	1	2	1	1	1	1	0	0	0	1	1	0	0	1	1	1	0	0	0	1	1	1	1	0	1	1	0	1	1	0	0	0	0	0	0
Gilliamella apis NO3	3	1	2	1	1	1	1	0	0	0	1	1	0	0	1	1	1	0	0	0	1	1	1	1	0	1	1	0	1	1	0	0	0	0	0	0
Gilliamella apis NO4	3	1	2	1	1	1	1	0	0	0	1	1	0	0	1	1	1	0	0	0	1	1	1	1	0	1	1	0	1	1	0	0	0	0	0	0
Gilliamella apicola NO5	4	1	3	1	1	1	2	0	0	0	1	1	0	0	1	1	1	1	0	0	1	1	1	1	1	1	1	0	1	1	0	0	0	0	0	0
Gilliamella apicola NO6	4	1	3	1	1	1	2	0	0	0	1	1	0	0	1	1	1	1	0	0	1	1	1	1	1	1	1	0	1	1	0	0	0	0	0	0
Gilliamella apicola NO8	4	1	3	1	1	1	2	0	0	0	1	1	0	0	1	1	1	1	0	0	1	1	1	1	1	1	1	0	1	1	0	0	0	0	0	0
Gilliamella apicola P46G	4	1	3	1	2	1	2	1	0	0	1	1	0	0	1	1	1	0	0	0	2	1	1	2	2	2	1	0	1	1	0	0	0	0	0	0
Gilliamella apicola P54G	4	1	3	1	1	1	2	0	0	0	1	1	0	0	1	1	1	1	0	0	1	1	1	1	1	1	1	0	1	1	0	0	0	0	0	0
Gilliamella apis P62G	3	1	2	1	1	1	1	0	0	0	1	1	0	0	1	1	1	0	0	0	1	1	0	1	0	1	1	0	1	1	0	0	0	0	0	0
Gilliamella apicola P83G	3	1	2	1	1	1	1	0	0	0	1	1	0	0	1	1	1	0	0	0	1	1	1	1	1	1	1	0	1	1	0	0	0	0	0	0
Bifidobacterium asteroides PRL2011	1	1	1	2	1	1	0	1	0	1	0	0	0	1	0	2	0	0	1	1	0	1	1	0	0	1	1	0	0	0	0	1	0	0	0	0
Gilliamella apicola SCGC AB-598-B02	0	0	2	1	1	0	0	0	0	0	1	0	0	0	0	1	1	1	0	0	1	1	1	1	0	1	1	0	1	1	0	0	0	0	0	0
Gilliamella apicola SCGC AB-598-I20	2	0	3	1	1	0	1	0	0	0	1	1	0	0	1	1	1	0	0	0	1	1	0	1	0	1	1	0	1	1	0	0	0	0	0	0
Gilliamella apis SCGC AB-598-P17	0	1	1	1	0	0	0	0	0	0	0	1	0	0	0	0	1	0	0	0	0	0	0	0	0	0	0	0	0	0	0	0	0	0	0	0
Gilliamella apicola wkB1	4	1	3	1	1	1	2	0	0	0	1	1	0	0	1	1	1	1	0	0	1	1	1	1	1	1	1	0	1	1	0	0	0	0	0	0
Lactobacillus sp wkB10	1	0	0	0	0	0	1	0	0	0	0	0	0	0	0	0	0	0	0	0	0	0	0	0	0	0	0	0	0	0	0	0	0	0	0	0
Snodgrassella alvi wkB332	1	1	1	1	1	1	0	1	1	0	1	0	0	0	0	1	1	0	0	1	1	1	1	1	1	1	1	1	0	0	0	0	0	0	0	0
Bifidobacterium sp wkB338	1	1	1	2	1	1	0	1	0	1	0	0	0	1	0	2	0	0	1	1	0	1	1	0	0	1	1	0	0	0	0	1	0	0	0	0
Snodgrassella alvi wkB339	1	1	1	1	1	1	0	1	1	0	1	0	0	0	0	1	1	0	0	1	1	1	1	1	1	1	1	1	0	0	0	0	0	0	0	0
Bifidobacterium sp wkB344	1	1	1	2	1	1	0	1	0	1	0	0	0	1	0	2	0	0	1	1	0	1	1	0	0	1	1	0	0	0	0	1	0	0	0	0
Gilliamella apicola wkB7	3	1	2	1	1	1	1	0	0	0	1	1	0	0	1	0	1	1	0	0	1	1	1	1	1	1	1	0	1	1	0	0	0	0	0	0
Lactobacillus sp wkB8	1	0	0	0	0	0	1	0	0	0	0	0	0	0	0	0	0	0	0	0	0	0	0	0	0	0	0	0	0	0	0	0	0	0	0	0
