# Supplementary material for: Community Dynamics in Structure and Function of Honey Bee Gut Bacteria in Response to Winter Dietary Shift
Source: mBio. 2022 Aug 29;13(5):e01131-22. doi: 10.1128/mbio.01131-22 (PMC9600256; doi:10.1128/mbio.01131-22)
Supplement: TABLE S5 [file mbio.01131-22-s0005.pdf]

Species	Strain	Host	Size Mbp	Contig	Accession
Bartonella apis	W7133	*Apis mellifera*	2.6	Complete	
Bartonella apis	BBC0122	*Apis mellifera*	2.91	Complete	CP015625
Bartonella apis	BBC0178	*Apis mellifera*	2.6	Complete	CP015820
Bartonella apis	BBC0244	*Apis mellifera*	2.64	Complete	CP015821
Bartonella apis	PEB0122	*Apis mellifera*	2.6	16	LXYU00000000
Bartonella apis	PEB0149	*Apis mellifera*	2.53	7	LXYT00000000
Bartonella apis	PEB0150	*Apis mellifera*	2.58	12	LXYS00000000
Bifidobacterium asteroides	DSM 20089	*Apis mellifera*	2.14	115	JDTU00000000
Bifidobacterium asteroides	PRL2011	*Apis mellifera. ligustica*	2.17	Complete	NC_018720
Bifidobacterium asteroides	ESL0170	*Apis mellifera*	2.18	7	NZ_QGLH00000000
Bifidobacterium asteroides	Hma3	*Apis mellifera*	2.25	16	KQ034040
Bifidobacterium asteroides	wkB338	*Apis mellifera*	2.19	49	NPOR00000000
Bifidobacterium asteroides	ESL0198	*Apis mellifera*	2.24	12	NZ_QGLJ00000000
Bifidobacterium asteroides	ESL0199	*Apis mellifera*	2.17	7	NZ_QGLK00000000
Bifidobacterium asteroides	ESL0200	*Apis mellifera*	1.93	16	NZ_QGLL00000000
Bifidobacterium coryneforme	Bma6	*Apis mellifera*	1.75	6	JXBX00000000
Bifidobacterium coryneforme	DSM 20216	*Apis mellifera*	1.74	67	JDUF00000000
Bifidobacterium indicum	ESL0197	*Apis mellifera*	1.72	6	NZ_QGLI00000000
Bifidobacterium indicum	LMG 11587 / DSM 20214	*Apis mellifera*	1.73	Complete	CP006018
Bifidobacterium asteroides	7101	*Apis mellifera*	2.12	19	AWUN00000000
Bifidobacterium asteroides	A11	*Apis mellifera*	2.18	51	AWUO00000000
Bifidobacterium asteroides	wkB344	*Apis mellifera*	2.12	27	NPOQ00000000
Gilliamella apicola	A-12-12	*Apis mellifera*	2.94	102	NARO00000000
Gilliamella apicola	A-1-24	*Apis mellifera*	3.14	157	MZNE00000000
Gilliamella apicola	A-2-24	*Apis mellifera*	3.14	129	MZNF00000000
Gilliamella sp. A-4-12	A-4-12	*Apis mellifera*	2.63	104	NASK00000000
Gilliamella apicola	A-7-12	*Apis mellifera*	3.14	91	NARP00000000
Gilliamella apicola	A-7-24	*Apis mellifera*	2.94	107	NARQ00000000
Gilliamella apicola	A8	*Apis mellifera*	3.05	226	MZNG00000000
Gilliamella apicola	A9	*Apis mellifera*	3.03	362	NARS00000000
Gilliamella apicola	A-8-12	*Apis mellifera*	2.93	110	NARR00000000
Gilliamella apicola	A-9-12	*Apis mellifera*	3.15	189	NART00000000
Gilliamella sp. AM1	AM1	*Apis mellifera*	2.44	120	NASL00000000
Gilliamella apicola	AM4	*Apis mellifera*	3.06	166	NARY00000000
Gilliamella apicola	AM6	*Apis mellifera*	3	655	MZNH00000000

Species	Strain	Host	Size Mbp	Contig	Accession
Gilliamella sp. A-TSA1	A-TSA1	*Apis mellifera*	2.47	79	NASO00000000
Gilliamella sp. A-TSA2	A-TSA2	*Apis mellifera*	2.46	72	NASP00000000
Gilliamella sp. A-TSA3	A-TSA3	*Apis mellifera*	2.47	77	NASQ00000000
Gilliamella sp. A-TSA4	A-TSA4	*Apis mellifera*	2.46	86	NASR00000000
Gilliamella apicola	AW11	*Apis mellifera*	2.94	328	NARX00000000
Gilliamella apicola	AW13	*Apis mellifera*	3.13	371	NARU00000000
Gilliamella apicola	Aw-17	*Apis mellifera*	2.93	130	MZNI00000000
Gilliamella apicola	B02	*Apis mellifera*	1.81	544	JAIM00000000
Gilliamella apis	ESL0169	*Apis mellifera*	2.43	13	NZ_QGLN00000000
Gilliamella apis	ESL0172	*Apis mellifera*	2.69	17	NZ_QGLO00000000
Gilliamella apicola	ESL0177	*Apis mellifera*	3.09	19	NZ_QGLP00000000
Gilliamella apicola	ESL0178	*Apis mellifera*	2.89	18	NZ_QGLQ00000000
Gilliamella apicola	ESL0182	*Apis mellifera*	3.54	31	NZ_QGLR00000000
Gilliamella apicola	I20	*Apis mellifera*	2.21	389	JAIN00000000
Gilliamella apicola	M1-2G	*Apis mellifera*	2.39	10	LZGQ00000000
Gilliamella apicola	M6-3G	*Apis mellifera*	2.71	85	MCIU00000000
Gilliamella apicola	N10	*Apis mellifera*	2.9	202	NARV00000000
Gilliamella apicola	N-12-12	*Apis mellifera*	3.08	147	NAHT00000000
Gilliamella apicola	N-15-12	*Apis mellifera*	3.05	264	NAHX00000000
Gilliamella apicola	N2	*Apis mellifera*	2.91	88	NARW00000000
Gilliamella apicola	N-22	*Apis mellifera*	2.97	92	NASB00000000
Gilliamella apicola	N4	*Apis mellifera*	2.91	127	NARN00000000
Gilliamella apicola	N6	*Apis mellifera*	2.91	82	NARZ00000000
Gilliamella sp. N-G1	N-G1	*Apis mellifera*	2.48	65	NASS00000000
Gilliamella sp. N-G2	N-G2	*Apis mellifera*	2.71	77	NAST00000000
Gilliamella sp. N-G3	N-G3	*Apis mellifera*	2.48	83	NASU00000000
Gilliamella sp. N-G4	N-G4	*Apis mellifera*	2.44	142	NASV00000000
Gilliamella apicola	N-G5	*Apis mellifera*	3.06	99	NASA00000000
Gilliamella sp. NO1	NO1	*Apis mellifera*	2.52	156	NASI00000000
Gilliamella apicola	NO10	*Apis mellifera*	3.05	398	NAHU00000000
Gilliamella sp. NO12	NO12	*Apis mellifera*	2.53	68	NASH00000000
Gilliamella sp. NO13	NO13	*Apis mellifera*	2.53	60	NASG00000000
Gilliamella sp. NO14	NO14	*Apis mellifera*	2.52	75	NASF00000000
Gilliamella sp. NO15	NO15	*Apis mellifera*	2.52	50	NASJ00000000
Gilliamella sp. NO16	NO16	*Apis mellifera*	2.53	57	NASE00000000

Species	Strain	Host	Size Mbp	Contig	Accession
Gilliamella sp. NO3	NO3	*Apis mellifera*	2.52	54	NASD00000000
Gilliamella sp. NO4	NO4	*Apis mellifera*	2.52	214	NASM00000000
Gilliamella apicola	NO5	*Apis mellifera*	3.06	143	NAHV00000000
Gilliamella apicola	NO6	*Apis mellifera*	3.06	129	NAHR00000000
Gilliamella apicola	NO8	*Apis mellifera*	3.04	529	NAHS00000000
Gilliamella sp. N-W3	N-W3	*Apis mellifera*	2.67	90	NASW00000000
Gilliamella apicola	P17	*Apis mellifera*	1.47	296	JAIO00000000
Gilliamella apicola	P46G	*Apis mellifera*	7.42	2779	MCIV00000000
Gilliamella apicola	P54G	*Apis mellifera*	3.11	55	LZGJ00000000
Gilliamella apicola	P62G	*Apis mellifera*	2.55	20	LZGI00000000
Gilliamella apicola	P83G	*Apis mellifera*	2.49	28	LZGH00000000
Gilliamella apicola	wkB1	*Apis mellifera*	3.14	Complete	CP007445
Gilliamella apicola	wkB7	*Apis mellifera*	2.9	Complete	LZGG01000000
Lactobacillus apinorum	Fhon13	*Apis mellifera*	1.46	33	JXCT00000000
Lactobacillus sp.	ESL0263	*Apis mellifera*	1.82	12	NZ_REHL00000000
Lactobacillus apis	R-53131	*Apis mellifera*	1.68	19	FMAN00000000
Lactobacillus sp.	wkB10	*Apis mellifera*	2.08	32	JRJB00000000
Lactobacillus kimbladii	Hma2	*Apis mellifera*	2.19	40	JXLH00000000
Lactobacillus kullabergensis	Biut2	*Apis mellifera*	2.12	37	JXBY00000000
Lactobacillus sp.	ESL0261	*Apis mellifera*	2.11	17	NZ_REHN00000000
Lactobacillus melliventris	ESL0184	*Apis mellifera*	2.04	4	NZ_QGLG00000000
Lactobacillus melliventris	Hma8	*Apis mellifera*	2.12	23	JXLI00000000
Lactobacillus sp.	ESL0260	*Apis mellifera*	1.93	22	NZ_REHO00000000
Lactobacillus helsingborgensis	Bma5	*Apis mellifera*	2.02	28	JXJR00000000
Lactobacillus sp.	wkB8	*Apis mellifera*	1.93	Complete	CP009531
Lactobacillus sp.	ESL0262	*Apis mellifera*	1.87	15	NZ_REHM00000000
Snodgrassella alvi	A12	*Apis mellifera*	2.4	214	NAGX00000000
Snodgrassella alvi	A5	*Apis mellifera*	2.43	120	NAHF01000000
Snodgrassella alvi	A-9-24	*Apis mellifera*	2.5	62	NAHH01000000
Snodgrassella alvi	ESL0196	*Apis mellifera*	2.45	15	NZ_QGLS00000000
Snodgrassella alvi	N-S2	*Apis mellifera*	2.42	73	NAHM01000000
Snodgrassella alvi	N-S4	*Apis mellifera*	2.42	38	NAHO00000000
Snodgrassella alvi	N-W7	*Apis mellifera*	2.42	62	NAHJ01000000
Snodgrassella alvi	wkB332	*Apis mellifera*	2.49	30	MEIJ00000000
Snodgrassella alvi	wkB339	*Apis mellifera*	2.5	27	MEII00000000

Species	Strain	Host	Size Mbp	Contig	Accession
Lactobacillus mellifer	Bin4	*Apis mellifera*	1.82	28	JXJQ00000000
Lactobacillus mellis	Hon2	*Apis mellifera*	1.81	17	JXBZ00000000
